# H_2_O_2_ self-supplying and GSH-depleting nanosystem for amplified NIR mediated-chemodynamic therapy of MRSA biofilm-associated infections

**DOI:** 10.1186/s12951-024-02350-6

**Published:** 2024-03-16

**Authors:** Yulan Zhao, Yang Wu, Quan Xu, Yi Liu, Zhiyong Song, Heyou Han

**Affiliations:** 1 National Key Laboratory of Agricultural Microbiology, College of Life Science and Technology, Huazhong Agricultural University, Wuhan, 430070 China; 2 National Key Laboratory of Agricultural Microbiology, College of Chemistry, Huazhong Agricultural University, Wuhan, 430070 China

**Keywords:** Fenton-like reaction, NIR mediated-chemodynamic therapy, Antibiofilms, Wound healing

## Abstract

Reactive oxygen species (ROS) has emerged as potent therapeutic agents for biofilm-associated bacterial infections. Chemodynamic therapy (CDT), involving the generation of high-energy ROS, displays great potential in the therapy of bacterial infections. However, challenges such as insufficient hydrogen peroxide (H_2_O_2_) and over-expressed glutathione (GSH) levels within the microenvironment of bacterial biofilms severely limit the antibacterial efficacy of CDT. Herein, we have developed a multifunctional nanoplatform (CuS@CaO_2_@Dex) by integrating copper sulfide (CuS) and calcium peroxide (CaO_2_) into dextran (Dex)-coated nanoparticles. This innovative platform enhanced ROS generation for highly efficient biofilm elimination by simultaneously supplying H_2_O_2_ and depleting GSH. The Dex-coating facilitated the penetrability of CuS@CaO_2_@Dex into biofilms, while CaO_2_ generated a substantial amount of H_2_O_2_ in the acidic biofilm microenvironment. CuS, through a Fenton-like reaction, catalyzed the conversion of self-supplied H_2_O_2_ into hydroxyl radicals (•OH) and consumed the overexpressed GSH. Additionally, the incorporation of near-infrared II (NIR II) laser irradiation enhanced the photothermal properties of CuS, improving the catalytic efficiency of the Fenton-like reaction for enhanced antibacterial effects. In vivo experiments have demonstrated that CuS@CaO_2_@Dex exhibited remarkable antibacterial and antibiofilm efficacy, exceptional wound healing capabilities, and notable biosafety. In summary, the Dex-coated nanoplatform proposed in this study, with its self-sterilization capability through ROS, holds significant potential for future biomedical applications.

**Supplementary Information:**

The online version contains supplementary material available at 10.1186/s12951-024-02350-6.

## Introduction

The emergence of drug-resistant bacteria, fueled by the widespread and indiscriminate use of antibiotics, has become a critical global challenge to human health [1, 2]. Especially, more than 80% bacterial infection diseases are attributed to bacterial biofilm, posing a severe threat to human life and health due to their high mortality rate and challenging recovery processes [3]. Bacterial biofilms manifest on bacterial surfaces as aggregates created by the envelopment of bacteria with extracellular polymers (EPS) produced by the bacteria. The EPS within biofilm acts as a guardian barrier, significantly enhancing bacterial tolerance to host immune defenses, environmental pressures, and antibiotics. In comparison to planktonic bacteria, biofilm-embedded bacteria exhibit a heightened resistance, resulting in a thousand-fold increase in bacterial resilience [4–7]. Consequently, there is an urgent need for novel antibiotic-free agents to effectively treat bacterial infection and eliminate mature biofilms.

In recent years, antibacterial strategies associated with reactive oxygen species (ROS) have drawn wide attentions and tremendous interests due to their ability to circumvent bacterial resistance [8, 9]. Previous research has shown that excess ROS can induce lipid peroxidation and direct damage proteins and DNA, ultimately leading to bacterial eradication [10–12]. Nanomaterials have been extensively explored for their capacity to generate ROS through various therapeutic modalities such as photodynamic therapy (PDT) [13, 14], sonodynamic therapy (SDT) [15, 16] and chemodynamic therapy (CDT) [17, 18]. Among these approaches, CDT has become a research hotspot due to the specificity of infected site by endogenous trigger to avoid normal tissue damage from oxidative damage and without the need for external energy input. Furthermore, well-designed CDT can generate high-toxic •OH from the less toxic H_2_O_2_ via the Fenton-like reaction, making it an ideal antibacterial strategy. Despite infected wounds exhibiting higher level of H_2_O_2_ (∼100 µM) than normal tissues, it remains insufficient for achieving desired efficacy of CDT [19–23]. The introduction of exogenous H_2_O_2_ during antibacterial treatment can potentially harm normal tissues. To address this limitation, recent research has proposed a self-supplying H_2_O_2_ through the Fenton-like reaction for antibacterial therapy, overcoming the challenge of substantial need for exogenous H_2_O_2_. Recently, metal peroxides nanoparticles, containing metal ions and peroxide groups, have emerged as promising sources of H_2_O_2_, reacting with H_2_O under acidic conditions to produce a substantial amount of H_2_O_2_ [21, 24, 25]. In the previous study, CaO_2_ have been applied to H_2_O_2_ self-supplying of CDT to boost the activity of biofilms eradication [5, 26].

The effectiveness of CDT is closely linked to the choice of Fenton catalysts. Among various Fenton catalysts, copper-based nanocatalysts demonstrate a superior ability to catalyze H_2_O_2_ to generate •OH, exhibiting significantly higher catalytic activity compared to iron-based nanocatalysts [27]. However, the limited conversion rate of Cu^2+^ to Cu^+^ poses a constraint on the efficiency of the Fenton-like reaction. On the other hand, glutathione (GSH), a natural antioxidant, is excessively expressed in infected wounds sites, inevitably scavenging ROS. Intriguingly, Cu^2+^ can be reduced to Cu^+^ by GSH, further enhancing the catalytic efficiency of CDT [28, 29]. Concurrently, GSH depletion may augment the bactericidal property of ROS by disrupting redox homeostasis. Fenton reaction is also temperature-dependent, and the potency of CDT is significantly heightened with elevated temperatures [30, 31]. Copper sulfide (CuS) serves as an ideal antibacterial agent due to its excellent photothermal conversion ability, leveraging strong near-infrared (NIR) absorbance widely applied to photothermal therapy of bacterial infections [32, 33]. Hence, we propose a therapeutic strategy that combines self-supplied H_2_O_2_ and GSH depletion, complemented by near-infrared-mediated chemokinetic therapy, to enhance the antibacterial effect.

Nevertheless, the poor penetration ability of antibacterial agents poses a significant challenge in effectively targeting bacterial biofilms. Additionally, certain nanoparticles, lacking stability under physiological condition, may result in adverse effects to healthy tissues [34]. Hence, there is an urgent needed for a few therapeutic approaches that improve biofilm penetration and enhance biocompatibility. Dextran, a polysaccharide secreted by bacterial, has been testified to infiltrate the biofilm and participate in bacterial aggregation and biofilm formation [34, 35]. Previous studies have highlighted that antibacterial agents coated with dextran not only effectively penetrate the biofilm but also do not compromise their intrinsic activity [35]. Furthermore, dextran coated antibacterial agents exhibit significantly improved stability in different solutions due to their highly amphiphilic and biocompatible properties [34, 36]. Consequently, the exceptional biocompatibility and penetrability properties of dextran provide an opportunity for the development of advanced antibiofilm agent.

**Scheme 1:**
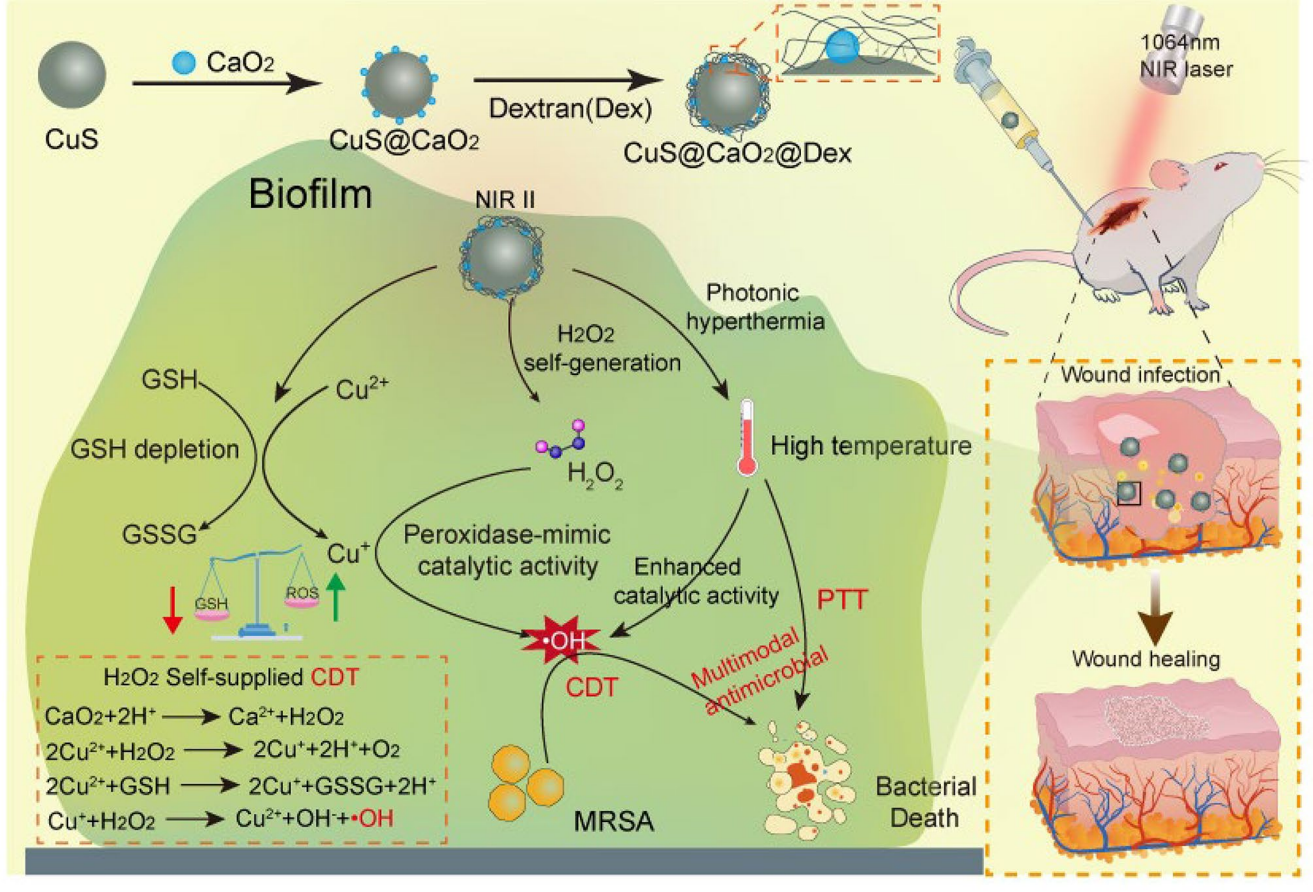
Schematic diagram for the synthesis of CuS@CaO_2_@Dex and its CDT/PTT synergistic therapy of biofilm-infected wounds

In this study, we present the design of a dextran-coated nanoplatform (CuS@CaO_2_@Dex) with a dual function of H_2_O_2_ self-supply and GSH consumption for synergistic CDT/PTT therapy of biofilms elimination. As depicted in Scheme 1, CaO_2_ is loaded on the surface of CuS, and dextran adhere to the surface of CuS@CaO_2_, resulting in the formation of CuS@CaO_2_@Dex. The introduction of dextran to CuS@CaO_2_@Dex not only facilitates penetration into biofilms but also enhances the biocompatibility of the nanoplatform. Within the acidic environment of biofilms, CaO_2_ reacts with H_2_O to produce H_2_O_2_, which is then catalyzed into highly cytotoxic •OH through a Fenton-like reaction. Simultaneously, CuS serves a dual role by depleting over-expressed GSH in infectious wound to disrupt redox homeostasis, and reducing Cu^2+^ to Cu^+^ through interaction with GSH, both of which enhance the catalytic efficiency of CDT. Moreover, the outstanding photothermal property of CuS further improves the catalytic efficiency of Fenton reaction under NIR II irradiation (1064 nm). Consequently, the proposed CuS@CaO_2_@Dex offers a robust strategy for H_2_O_2_ self-supply and GSH-depletion, achieving synergistic antibacterial performance in CDT/PTT and demonstrating tremendous potential in the treatment of biofilm-associated bacterial infections.

## Experimental section

### Materials

Polyvinylpyrrolidone (PVP), dextran (20 kDa), sodium sulfide nonahydrate (Na_2_S·9H_2_O), 5,5’-Dithiobis (2-nitrobenzoic acid) (DTNB), copper (II) chloride dihydrate (CuCl_2_·2H_2_O), propidium iodide (PI), glutathione (GSH) and ammonia solution (NH_3_·H_2_O, 28–30%) were purchased from Aladdin-Reagent. Hydrazine hydrate (85%), calcium chloride (CaCl_2_) and hydrogen peroxide (H_2_O_2_, 30%) were bought by Sinopharm Chemical Reagent. Hydrogen peroxide assay kit, 2′,7′-dichlorofluorescin diacetate (DCFH-DA) and 2-(4-amidinophenyl)-6-indolecarbamidine dihydrochloride (DAPI) were supplied by Beyotime Biotechnology. Roswell Park Memorial Institute 1640 Medium (RPMI 1640 Medium), Penicillin-Streptomycin Solution, and fetal bovine serum (FBS) were purchased from Solarbio Reagent.

### Synthesis of CuS and CaO_2_

The synthesis of CuS was performed following the procedure outlined in a previous literature reference [37]. Briefly, 480 mg of PVP was added to a 50 mL of CuCl_2_ solution (0.02 mol L^− 1^). Next, 12 µL of hydrazine hydrate was mixed with the above solution under continuous stirring, followed by the addition of Na_2_S solution (400 µL, 320 µg mL^− 1^). The resulting mixture was then heated to 65 ℃ and allowed to react under stirring for 2 h. The final precipitates were obtained by centrifugation and subsequently rinsed several times with deionized water. Spherical aggregates of CaO_2_ were fabricated based on previous reports [38]. Briefly, 15 mg of CaCl_2_ and 350 mg of PVP were separately dispersed in 15 mL ethanol under ultrasound treatment. Subsequently, 1 mL 0.8 mol L^− 1^ NH_3_·H_2_O was mixed with the above solution. Then, 200 µL of 1 mol L^− 1^ H_2_O_2_ was introduced at the speed of 50 µL min^− 1^ through an injection pump. Finally, the products were collected by centrifugation (14,000 rpm, 20 min) and then wash thrice with ethanol.

### Synthesis and characterization of CuS@CaO_2_@Dex

The synthesis of CuS@CaO_2_@Dex involved a straightforward mixing and stirring process. Briefly, 1 mg of CuS and 1 mg of CaO_2_ were individually dissolved in 5 mL dimethyl sulfoxide (DMSO). After 12 h, 1 mg of dextran was dissolved in the above solution and stirred overnight. Subsequently, the obtained precipitates were obtained by centrifugation and then washed thrice with DMSO. The purified CuS@CaO_2_@Dex was stored in 4 ℃ for the subsequent characterizations. The morphologies and structure of materials were further analyzed via transmission electron microscopy (TEM, JEOL, JEM-2010, Japan). The zeta potential and hydrodynamic sizes of CuS@CaO_2_@Dex nanocomposites were measured using Malvern Zeta sizer (Nano-ZS 900) system.

### Photothermal effect of CuS@CaO_2_@Dex

The photothermal behavior of CuS@CaO_2_@Dex was examined using a digital NIR photothermal imaging system. Initially, various concentrations (25–400 µg mL^− 1^) CuS@CaO_2_@Dex was subjected to 1064 nm NIR laser irradiation (0.75 W cm^− 2^) for 5 min, and then the solution temperatures were recorded every 1 min. Subsequently, the photothermal effect of CuS@CaO_2_@Dex (200 µg mL^− 1^) was investigated varying power densities (0.25-1.0 W cm^− 2^) of NIR laser. Besides, the photostability of CuS@CaO_2_@Dex (200 µg mL^− 1^) was examined by repetitively toggling the NIR laser (0.75 W cm^− 2^) five times. The photothermal conversion efficiency of CuS@CaO_2_@Dex was calculated based on the temperature variation during the cooling phase using the following formula:

$$\eta =\frac{h\text{S}({T}_{max}-{T}_{{max}^{H2O}})}{I(1-{10}^{-A\lambda })}$$

$$h\text{S}=\frac{mCp}{{\uptau }\text{s}}$$

$$\theta =\frac{T-{T}_{surr}}{{T}_{max}-{T}_{surr}}$$

$$t=-\tau s\text{ln}\left(\theta \right)$$

In the above equation, *T*_*max*_ is the highest temperature of CuS@CaO_2_@Dex. ${T}_{{max}^{H2O}}$ the highest temperature of H_2_O. *I* stand for the laser power. *S* stands for the heated surface area. *A*^*λ*^ is the absorbance of CuS@CaO_2_@Dex solution at 1064 nm in the UV–vis spectrum. *h* stands for heat transfer coefficient. *η* stands for the photothermal conversion efficiency.

### H_2_O_2_ released from CaO_2_

20 µg CaO_2_ was dissolved in 1 mL PBS at two different pH levels (pH 6.0 and 7.4). The solutions were then shaken in a shaker at room temperature for 1 h. Next, the obtained samples (50 µL) were mixed with hydrogen peroxide detection reagent (100 µL) and allowed to react for 1 h. The absorbance at 570 nm was determined, and then the amount of H_2_O_2_ generated by CaO_2_ was calculated.

### Detection of the •OH

The generation of •OH by various nanomaterials treatments was assessed using 3,3′,5,5′-tetramethylbenzidine (TMB). Briefly, CaO_2_ and CuS@CaO_2_@Dex (300 µg mL^− 1^) at pH 6.0 were mixed with an equal volume of 1.25 mM TMB, followed incubation at 37 ℃ for 2 h. The changes in absorbance of the oxidized TMB (oxTMB) by •OH was then measured at 650 nm. Additionally, the •OH generation of CuS@CaO_2_@Dex (pH 6.0 and 7.4) under NIR irradiation or without NIR exposure as evaluated separately. Furthermore, the •OH generation of CuS@CaO_2_@Dex (pH 6.0 and 7.4) at specific time points was investigated by recording the absorbance at 650 nm of oxTMB. Moreover, we also used methylene blue (MB) to measure •OH generation. The different concentrations of CuS@CaO_2_@Dex reacted with the equal volume of MB (10 µg mL^− 1^) for 30 min and then the absorbance of MB at 665 nm was detected. The •OH generation was further measured through electron spin resonance (ESR) spectroscopy with 5, 5-Dimethyl-1-pyrroline N-oxide (DMPO) as a spin trap.

### GSH depletion

The GSH depletion capability of CuS@CaO_2_@Dex was assessed using Ellman’s assay [39]. Initially, 150 µg mL^− 1^ of CuS@CaO_2_@Dex were mixed with GSH (1 mM). After various reaction times, 100 µL of the resulting mixture was added into 890 µL PBS (pH 7.4), and followed by the introduction of 10 µL of DTNB (1 mM). The absorbance of the mixture solution was then obtained at 407 nm. The GSH-depleting properties of CaO_2_, CuS and CuS@CaO_2_@Dex were also explored at 1 h. The remaining GSH content in the reaction solution was determined using the standard curve of GSH.

### In vitro antibacterial assays

We assessed the antibacterial efficacy of CuS@CaO_2_@Dex against methicillin-resistant *Staphylococcus aureus* (MRSA) using the lysogeny broth (LB) agar plates counting method. Briefly, various concentrations of CuS@CaO_2_@Dex (25–150 µg mL^− 1^) were combined with MRSA (1 × 10^8^ CFU mL^− 1^) and simultaneously subjected to 5 min of 1064 NIR laser exposure (0.75 W cm^− 2^). After being incubated for 2 h at 37 °C, the mixed samples were diluted with PBS by a factor of 10,000, and then 50 µL bacterial suspension was then extracted for spreading on the LB agar plate at 37 °C overnight. Subsequently, bacterial colonies on the agar plate were photographed and colonies counting was performed for the bacterial viability. Besides, MRSA (1 × 10^8^ CFU mL^− 1^) was mixed with diverse nanoparticles of various treatment (PBS, PBS + NIR, CuS, CuS + NIR, CuS@CaO_2_@Dex + pH 7.4, CuS@CaO_2_@Dex + pH 6.0, CuS@CaO_2_@Dex + pH 7.4 + NIR, CuS@CaO_2_@Dex + pH 6.0 + NIR). The used concentrations of CaO_2_, CuS@CaO_2_, and CuS@CaO_2_@Dex were 46.8 µg mL^− 1^, 130.2 µg mL^− 1^, and 150 µg mL^− 1^, respectively. Subsequently, the bacterial survival rate was counted by the plate counting method. Moreover, the in vitro antibacterial properties of CuS@CaO_2_@Dex were also evaluated by live/dead bacterial staining with a fluorescence microscope. Briefly, MRSA (1 × 10^8^ CFU mL^− 1^) was processed with the same treatments described above. Subsequently, the mixed solution was incubated for 2 h, followed by being centrifuged to obtain MRSA. Next, 10 µL PI and 30 µL DAPI were added to bacterial solution away from light for 30 min and observed by confocal laser scanning microscopy (CLSM).

### Exploration of antibacterial mechanism

The morphology of MRSA by various treatments was observed using TEM and SEM. Briefly, MRSA suspensions (1 × 10^8^ CFU mL^− 1^) were processed with diverse treatments. Next, MRSA was fixed with 2.5% glutaraldehyde for 4 h at room temperature, and next dehydrated by varying concentrations of ethanol solutions (30–100%) for 10 min. Finally, the dehydrated MRSA in ethanol was dropped on the copper grid and observed by TEM and SEM. The intracellular ROS of MRSA was measured by 2’,7’-dichlorodihydrofluorescein diacetate (DCFH-DA). In brief, MRSA suspensions (1 × 10^8^ CFU mL^− 1^) were mixed with varying nanomaterials. Subsequently, the mixture was incubated for 5 min, both with and without NIR irradiation. Next, 50 µL DCFH-DA (20 µM) was introduced to MRSA for 30 min incubation and observed via a fluorescence microscope to detect the generation of ROS. In addition, the lipid peroxidation of MRSA treated with different nanomaterials was explored by measuring the level of intracellular malondialdehyde (MDA).

### In vitro antibiofilm properties

The in vitro antibiofilm property of CuS@CaO_2_@Dex was investigated. For this purpose, 10 µL of MRSA suspension (1 × 10^8^ CFU mL^− 1^) was introduced to the 190 µL of TSB medium in the 96-well plate, followed by being cultured at 37 ℃ for two days for the formation of mature biofilms. Subsequently, various nanomaterials by different treatment (PBS, PBS + NIR, CuS, CuS + NIR, CuS@CaO_2_@Dex + pH 7.4, CuS@CaO_2_@Dex + pH 6.0, CuS@CaO_2_@Dex + pH 7.4 + NIR, CuS@CaO_2_@Dex + pH 6.0 + NIR) were poured into the 96-well plates. The NIR laser (0.75 W cm^− 2^) was utilized to irradiate the above samples for 5 min and then incubated for 12 h. Next, 0.5% crystal violet was applied to the mixture for 30 min incubation and then the treated biofilms were washed with PBS to wash out excess dye. Finally, the stained biofilm was dissolved in 150 µL ethanol solution and then the absorbance at 590 nm were detected by a microplate reader. Afterwards, CLSM was be employed to further assess the antibiofilm activity of CuS@CaO_2_@Dex. Briefly, after forming the mature biofilm, various nanoparticles including CaO_2_, CuS, CuS@CaO_2_ and CuS@CaO_2_@Dex were introduced into the biofilm wells and then irradiated by NIR laser (0.75 W cm^− 2^) for 5 min. After 24 h of incubation, PI and DAPI were added to the above samples and then cultured away from light for 30 min. Finally, MRSA biofilms were visualized by CLSM.

### In vivo anti-infective assays

All the animal experiments were performed and adhered to a protocol approved by the Animal Care and Use Committee of Huazhong Agriculture University (Ethical ID number: HZAUMO-2023–0194). We fabricated the wound infection model using female BALB/c mice (6–8 weeks) to investigate in vivo antibiofilm performance of CuS@CaO_2_@Dex. Briefly, a circular skin defect (about 1 cm in diameter) was cut out with scissors on the shaved dorsal area. After that, MRSA suspension (100 µL, 1 × 10^8^ CFU mL^− 1^) was added to the wound skin via hypodermic injection and cultured for 48 h to eventually form MRSA biofilm infection model. Afterwards, mice were randomly assigned to seven groups (*n* = 5): control PBS, PBS + NIR, CaO_2_, CuS@CaO_2_, CuS@CaO_2_ + NIR, CuS@CaO_2_@Dex, CuS@CaO_2_@Dex + NIR. The used concentrations of CaO_2_, CuS@CaO_2_, and CuS@CaO_2_@Dex were 46.8 µg mL^− 1^, 130.2 µg mL^− 1^, and 150 µg mL^− 1^, respectively. 50 µL of the various nanomaterials were shifted to the wound sites. After 30 min of penetration into the biofilm, the infected sites were treated with 5 min of 1064 NIR laser exposure (0.75 W cm^− 2^). Then, the infected wounds were taken pictures and the mice weights were monitored on days 0, 1, 3, 5 and 7. On days 7, a medical cotton swab was served to dip the bacteria of the infected wound and then a spread plate was implemented to investigate the amounts of bacteria at the wound. At the last period of the treatment process, these mice were killed and then the skin wounds were excised. Next, the harvested skins were immersed in 4% paraformaldehyde for hematoxylin and eosin (H&E) and Masson staining. The primary organs (heart, liver, spleen, lung, and kidney) were excised for H&E staining analysis. Additionally, blood samples were extracted for the blood biochemistry indexes to estimate the in vivo biosafety of CuS@CaO_2_@Dex.

### Cytotoxicity testing

Human normal liver cell LO_2_ were utilized to investigate the cytotoxicity of CuS@CaO_2_@Dex by methyl thiazolyl tetrazolium (MTT) assays. Firstly, LO_2_ were carefully grow on in 96-well plates for 24 h. Subsequently, diverse concentrations of CuS@CaO_2_@Dex (0, 25, 50, 100, 150 µg mL^− 1^) were put in each well with 5 min of NIR irradiation (0.75 W cm^− 2^) for 6 h incubation. 20 µL MTT reagent (5 mg mL^− 1^) was placed in each well for a 3 h of incubation. Next, 150 µL DMSO solution replaced the culture medium in each well. 100 µL of the above supernatant was applied to determine the absorbance at 490 nm through a microplate reader. All experiments were done in six replicates (*n* = 6). Besides, the cytotoxicity of CuS@CaO_2_@Dex was investigated by the hemolytic assay. The obtained red blood cell (RBC) was processed with the diverse concentrations of CuS@CaO_2_@Dex (0, 25, 50, 100, 150, 200 µg mL^− 1^) for an incubation of 2 h. As controls, the RBC treated with ultrapure water and PBS were also performed.

The safety and migration ability of CuS@CaO_2_@Dex on fibroblasts was evaluated using a transwell migration assay. Briefly, CuS@CaO_2_@Dex (150 µg mL^− 1^, 2 mL) was introduced to the damaged fibroblasts solution treated with lipopolysaccharide (LPS) and then irradiated by NIR irradiation or not. After 24 h of incubation, fibroblasts were digested by trypsin and resuspended with in serum-free medium. Subsequently, fibroblasts suspension (2 × 10^4^) was seeded in the top chamber. The lower well was added to media supplemented with 10% FBS and cultured for a day. After that, 0.5% crystal violet solution was used to treat fibroblasts in the top chamber and then the cells on the other side of the membranes were erased with medical cotton swabs to clear unmigrated cells. Finally, migrated fibroblasts were observed and photographed by a microscope. The damaged fibroblasts and normal fibroblasts were acted as the negative and positive controls, respectively.

### Statistical analysis

The obtained results were expressed as the standard deviations (SDs) above and below the mean. The statistical analysis was carried out with the Student’s t-test (Origin 9.0 software). A *p*-value < 0.05 was considered statistically significant.

## Results and discussion

### Synthesis and characterization of CuS@CaO_2_@Dex

**Fig. 1:**
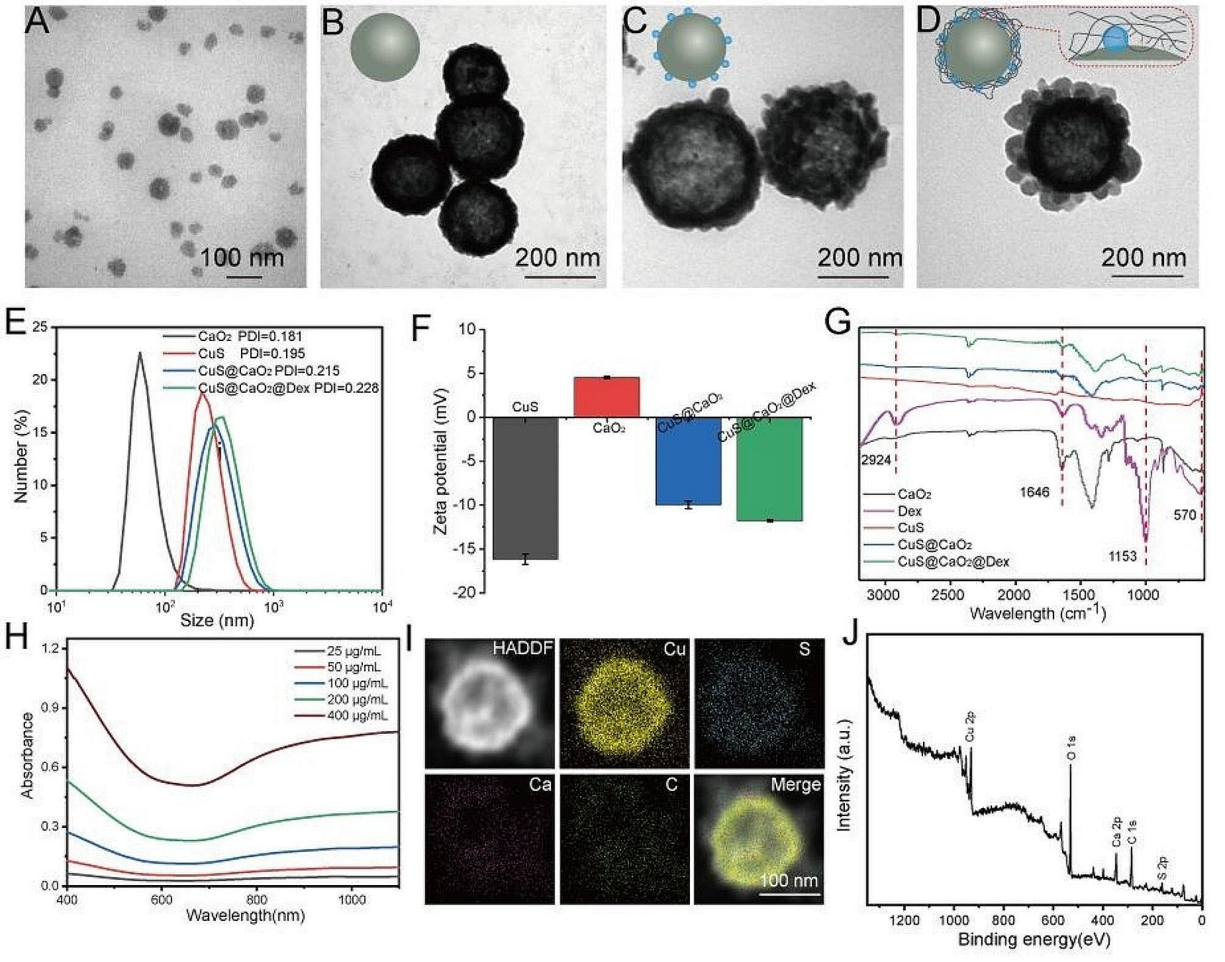
The characterization of CuS@CaO_2_@Dex. The TEM pictures of (**A**) CaO_2_, (**B**) CuS, (**C**) CuS@CaO_2_ and (**D**) CuS@CaO_2_@Dex. (**E**) Hydrodynamic size, (**F**) Zeta potential and (**G**) FTIR spectra of CaO_2_, CuS, CuS@CaO_2_, and CuS@CaO_2_@Dex. (**H**) UV–vis spectra of different concentrations of CuS@CaO_2_@Dex. (**I**) Elemental mapping of Cu, S, Ca and C in CuS@CaO_2_@Dex. (**J**) XPS spectra of CuS@CaO_2_@Dex.

As illustrated in Scheme 1, the preparation of CuS@CaO_2_@Dex involved the integration of CaO_2_ and dextran on the surface of CuS. Initially, CaO_2_ was synthesized using a wet chemical method [38]. TEM revealed the uniformly dispersed and spherical morphology of CaO_2_ (Fig. 1A). The hydration particle size (DLS) of CaO_2_ exhibited the size of 104.5 ± 1 nm (Fig. 1E) and the surface charge of CaO_2_ was measured as 4.5 ± 0.1 mV by zeta potential (Fig. 1F). Subsequently, CuS was successfully obtained through an ion-exchanging process [37]. The TEM image presented a hollow mesoporous spherical morphology for CuS (Fig. 1B). The DLS size was measured as 283.6 ± 5.9 nm (Fig. 1E) and zeta potential indicated a surface charge of -16.2 ± 0.6 mV (Fig. 1F). Following this, CaO_2_ was adsorbed onto the surface of CuS to form CuS@CaO_2_ through electrostatic absorption. TEM demonstrated unevenly distributed CaO_2_ on the surface of CuS (Fig. 1C). Its particle size was 331.0 ± 7.2 nm, confirming the successful adsorption of CaO_2_ (Fig. 1E). As expected, the surface potential of CuS@CaO_2_ also increased, indicating the adsorption of CaO_2_ on the surface of CuS (Fig. 1F). Finally, as shown in Fig. 1D, dextran was coated on CuS@CaO_2_ through the coordination of Ca^2+^ and Cu^2+^ with the hydroxyl group to enhance the stability of CuS@CaO_2_ (Fig. S1) [36]. The hydrodynamic size of CuS@CaO_2_@Dex was higher than that of CuS@CaO_2_, which was related to the successful encapsulation of dextran on the material surface (Fig. 1E).

The chemical structures of the prepared CaO_2_, CuS, dextran, CuS@CaO_2_ and CuS@CaO_2_@Dex were characterized by FTIR spectroscopy. The absorption peaks of CaO_2_ around 570 cm^− 1^ were attributed to O − Ca − O vibration, and 1646 cm^− 1^ attributed to the stretching mode of the C = O bond of PVP. Compared to CuS, the absorption peaks of CaO_2_ were evident in the spectrum of CuS@CaO_2_ and CuS@CaO_2_@Dex, indicating the modification of CaO_2_ on the surface of CuS (Fig. 1G). Strong peaks at 2924 cm^− 1^ and 1153 cm^− 1^ in the spectra of CuS@CaO_2_@Dex corresponded to the stretching of the C − H bond and the C − O−C vibration of the glycosidic bridge between saccharide units, respectively, affirming the successful introduction of dextran [40]. The UV spectrum (Fig. 1H) indicated that CuS@CaO_2_@Dex exhibited a strong NIR absorption property with a concentration-dependent manner, suggesting superior photothermal conversion performance. TEM mapping pictures revealed the presence of Cu, S, Ca and C elements in nanoparticles, confirming the successful formation of CuS@CaO_2_@Dex (Fig. 1I). Furthermore, the elemental composition and chemical bonds of CuS@CaO_2_@Dex were investigated by X-ray photoelectron spectroscopy (XPS). The survey spectrum of CuS@CaO_2_@Dex displayed the specific peaks corresponding to Ca, O, Cu, S and C elements, consistent with elemental mapping results (Fig. 1J). High-resolution spectrum of Cu 2p showed major peaks at 932.5 eV (Cu 2p3/2) and 952.2 eV (Cu 2p1/2), the specific peak of S 2p at 163.1 eV and the Ca 2p peaks at 347.83 eV (Ca 2p3/2) and 351.7 eV (Ca 2p1/2), further confirming the successful synthesis of CuS@CaO_2_@Dex (Fig. S2).

### Photothermal property of CuS@CaO_2_@Dex

**Fig. 2:**
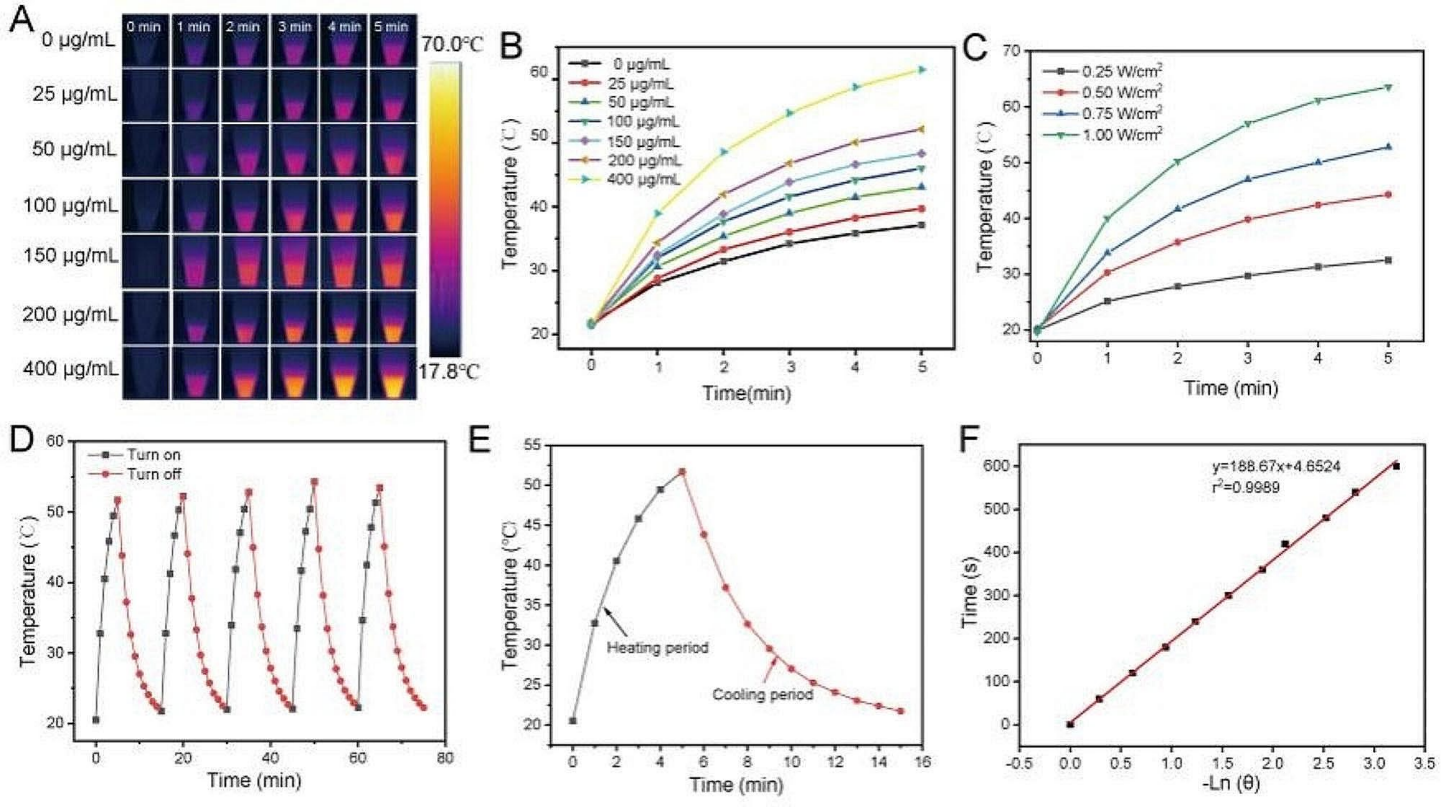
Photothermal performance of CuS@CaO_2_@Dex. (**A**) Infrared thermographic images of CuS@CaO_2_@Dex. (**B**) Temperature elevation of CuS@CaO_2_@Dex with diverse concentrations under the action of laser irradiation (1064 nm, 0.75 W cm^− 2^). (**C**) Temperature elevation of CuS@CaO_2_@Dex (200 µg mL^− 1^) at varying laser power densities. (**D**) photostability of CuS@CaO_2_@Dex (200 µg mL^− 1^) by repeatedly adjusting the switch of laser for five times. (**E**) The temperature variation curve of single heating-cooling for CuS@CaO_2_@Dex (200 µg mL^− 1^, 0.75 W cm^− 2^). (**F**) The detection of time constant for CuS@CaO_2_@Dex via the curve during the cooling period

We systematically investigated the in vitro photothermal capacity of CuS@CaO_2_@Dex with the aid of 1064 nm NIR laser irradiation. Initially, the photothermal abilities of CuS@CaO_2_@Dex at various concentrations were examined. As depicted in Fig. 2A and B, CuS@CaO_2_@Dex exhibited distinct thermal imaging effect, with varying degrees of temperature increase corresponding to different concentrations under 0.75 W cm^− 2^ laser irradiation for 5 min. Remarkably, the solution temperature of CuS@CaO_2_@Dex (200 µg mL^− 1^) rapidly increased from 21.8 to 52.2 °C within 300 s. Additionally, the impact of various laser power densities on the solution temperature of CuS@CaO_2_@Dex was evaluated. Specifically, the solution temperatures of CuS@CaO_2_@Dex (200 µg mL^− 1^) reached 52.8 °C (0.75 W cm^− 2^, 5 min) and 63.6 °C (1.00 W cm^− 2^, 5 min), respectively (Fig. 2C and S4). These results illustrated the outstanding photothermal conversion capacity of CuS@CaO_2_@Dex. Subsequently, the photostability of CuS@CaO_2_@Dex was investigated by repeatedly adjusting the switch of the NIR laser (200 µg mL^− 1^, 0.75 W cm^− 2^). As observed in Fig. 2D, the highest temperature of CuS@CaO_2_@Dex exhibited negligible variation after repeatedly adjusting the switch of 1064 nm laser for five times, demonstrating the high photostability of CuS@CaO_2_@Dex. According to the photothermal heating-cooling curves (Fig. 2E), we determined the photothermal conversion efficiency (PTCE, *η*) of CuS@CaO_2_@Dex. The *η* was calculated using formulas 1–4 as 16.8% (Fig. 2F). (*T*_*max*_ = 51.7 ℃, *T*_*max*,_*H*_*2*_*O* = 35.3 ℃, *T*_*sur*_ = 20.5 ℃, *A*^*λ*^ = 0.374, *m* = 200 mg, *Cp* = 4.2 [J kg^− 1^·℃^−1^]). These results strongly demonstrated that CuS@CaO_2_@Dex was effective PTT agents and is anticipated to be a promising choice for medical applications.

### Self-cascading ROS generation

**Fig. 3:**
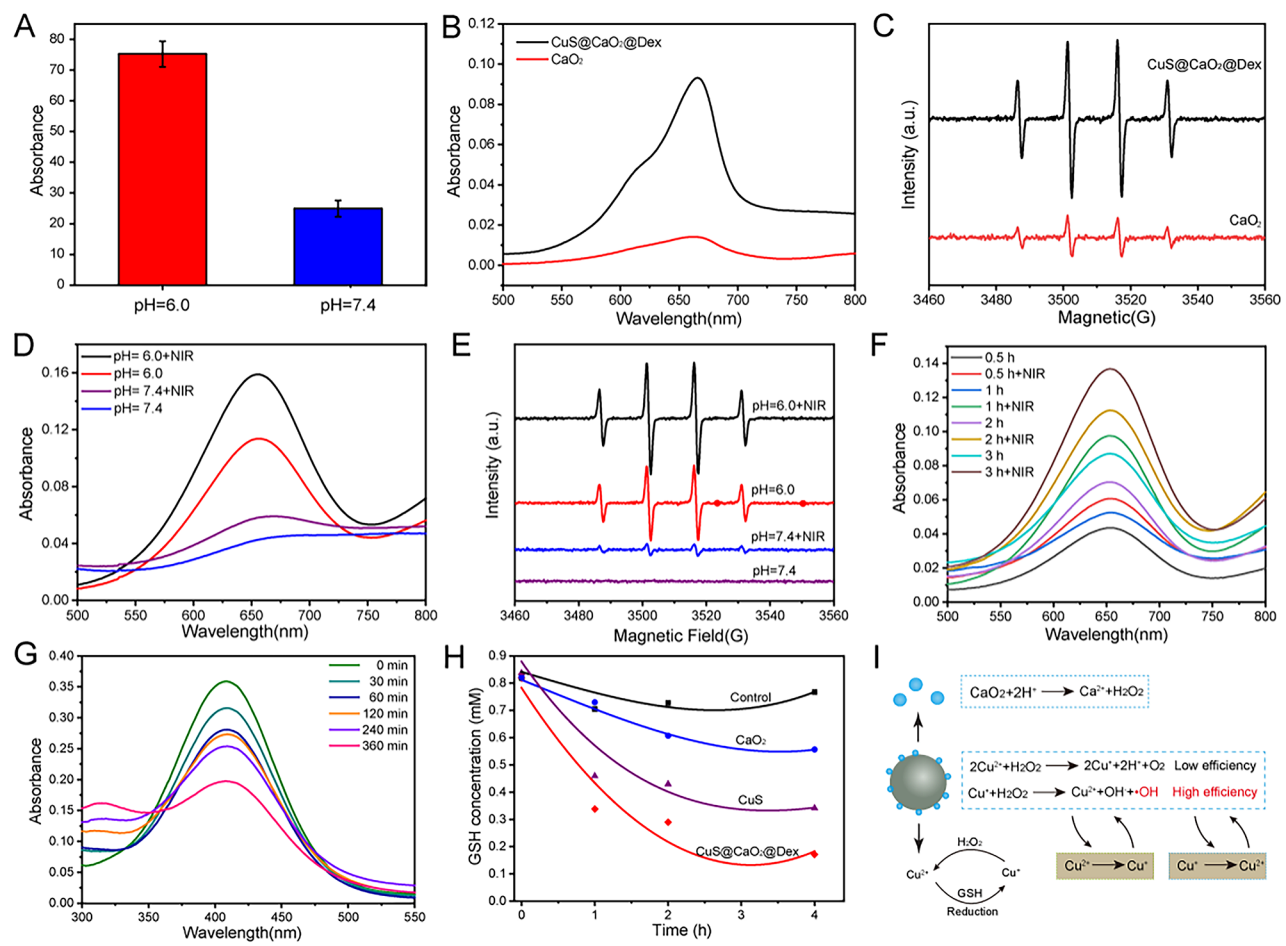
The •OH generation and GSH depletion of CuS@CaO_2_@Dex. (**A**) H_2_O_2_ production of CaO_2_ in different pH value. (**B**) and (**D**) The •OH generation determined by TMB after various treatments. (**C**) and (**E**) The generation of •OH determined by ESR after diverse treatments. (**F**) The •OH generation of CuS@CaO_2_@Dex at different time points with various treatments. (**G**) GSH-depleting properties of CuS@CaO_2_@Dex at various time points by DTNB. (**H**) The GSH-depleting capacities of CuS, CaO_2_, and CuS@CaO_2_@Dex. (**I**) Schematic illustration of CuS@CaO_2_@Dex self-sufficient H_2_O_2_ and GSH mediated reduction of Cu^2+^ to Cu^+^ under the action of 1064 nm NIR laser irradiation

We further simulated the infective microenvironment to explore the function of the nanomaterial. Firstly, we investigated the ability of CaO_2_ to generate H_2_O_2_ under acidic condition using hydrogen peroxide detection kit. As depicted in Fig. 3A, CaO_2_ produced more H_2_O_2_ under slightly acidic conditions (pH 6.0, simulated infection microenvironment) than under neutral conditions. Next, we explored the •OH generation rate of CuS@CaO_2_@Dex under acidic conditions through a Fenton reaction. The •OH produced by CuS@CaO_2_@Dex could react with colorless TMB to generate the oxTMB with a major peak at 650 nm. As shown in Fig. 3B, H_2_O_2_ produced by CuS@CaO_2_@Dex was catalyzed to generate •OH due to the catalysis of Cu^2+^/Cu^+^. Furthermore, •OH generation was also investigated by electron spin resonance (ESR) spectra. CuS@CaO_2_@Dex could produce more •OH than CaO_2_ alone, owing to the absence of copper catalysis (Fig. 3C). The results of MB degradation experiment showed that •OH generation from CuS@CaO_2_@Dex exhibited a concentration-dependent change (Fig. S5).

Next, we evaluated the effect of NIR laser on •OH generation by CuS@CaO_2_@Dex. As shown in Fig. 3D, the absorption peak of oxTMB by CuS@CaO_2_@Dex was significantly enhanced after exposure to NIR light in the PBS (pH 6.0). Nevertheless, there was only a slight difference in the absorption peak of oxTMB in the PBS (pH 7.4), mainly due to the insufficient generation of H_2_O_2_. Furthermore, ESR further demonstrated that NIR laser could boost the catalytic efficiency of the Fenton reaction. The results obtained by ESR spectra were consistent with the earlier findings (Fig. 3D). These results indicated that CuS@CaO_2_@Dex could generate sufficient H_2_O_2_ under acidic conditions, and its catalytic efficiency could be significantly enhanced by NIR irradiation. Subsequently, we investigated •OH produced at different time points by the TMB assay. As presented in Fig. 3F, the absorption peak of oxTMB by CuS@CaO_2_@Dex gradually intensified with increasing time under acidic condition. Notably, the absorption peak of oxTMB was significantly enhanced after exposure of CuS@CaO_2_@Dex to NIR light. These results indicated that NIR irradiation, through the photothermal effect, strongly enhanced the catalytic activity of CuS@CaO_2_@Dex.

Glutathione (GSH) is a crucial intracellular antioxidant that maintains intracellular redox homeostasis and protects cellular components from damage. Endogenous GSH has the inherent capability to scavenge ROS, thereby mitigating the antibacterial effect of ROS-based antibacterial nanomaterials. The consumption of GSH can enhance the bactericidal effect of CDT by destroying redox homeostasis. Additionally, GSH could reduce Cu^2+^ to Cu^+^, further boosting the catalytic efficiency of CDT and augmenting the antibacterial properties (Fig. 3I). Consequently, we assessed the GSH depletion ability of CuS@CaO_2_@Dex using Ellman’s assay. The colorless DTNB reacted with GSH to generate a yellow product with distinctive absorption at 407 nm. The remaining GSH concentration for CuS@CaO_2_@Dex (pH 6.0) at various time points (0, 30, 60, 120 and 360 min) was determined by measuring the absorbance at 407 nm. The absorbance gradually decreased with the time increasing, indicating that CuS@CaO_2_@Dex led to sustained reduction in GSH concentration (Fig. 3G). After excluding GSH consumption from CaO_2_, both CuS and CuS@CaO_2_@Dex caused a decline in GSH content, confirming that Cu^2+^ ions of CuS@CaO_2_@Dex could remarkably consume GSH via a reduction reaction (Fig. 3H).

### In vitro antibacterial effect of CuS@CaO_2_@Dex

Encouraged by the favorable photothermal activity and ROS generation capacity of CuS@CaO_2_@Dex, we investigated its antibacterial activity using MRSA as the bacteria model. To elucidate the concentration-dependent antibacterial effect, various concentrations of CuS@CaO_2_@Dex were incubated with MRSA. Subsequently, the mixed samples underwent 5 min of NIR laser irradiation (0.75 W cm^− 2^), and the viability of MRSA was determined by plate counting method. The number of colonies significantly decreased with an increase in the concentration of CuS@CaO_2_@Dex (Fig. S7). Notably, when the concentration of CuS@CaO_2_@Dex was elevated to 150 µg mL^− 1^, the survival rate of MRSA was reduced to 4.84% ± 0.37%. Therefore, a concentration of 150 µg mL^− 1^ CuS@CaO_2_@Dex was selected for subsequent antibacterial experiments against MRSA.

**Fig. 4:**
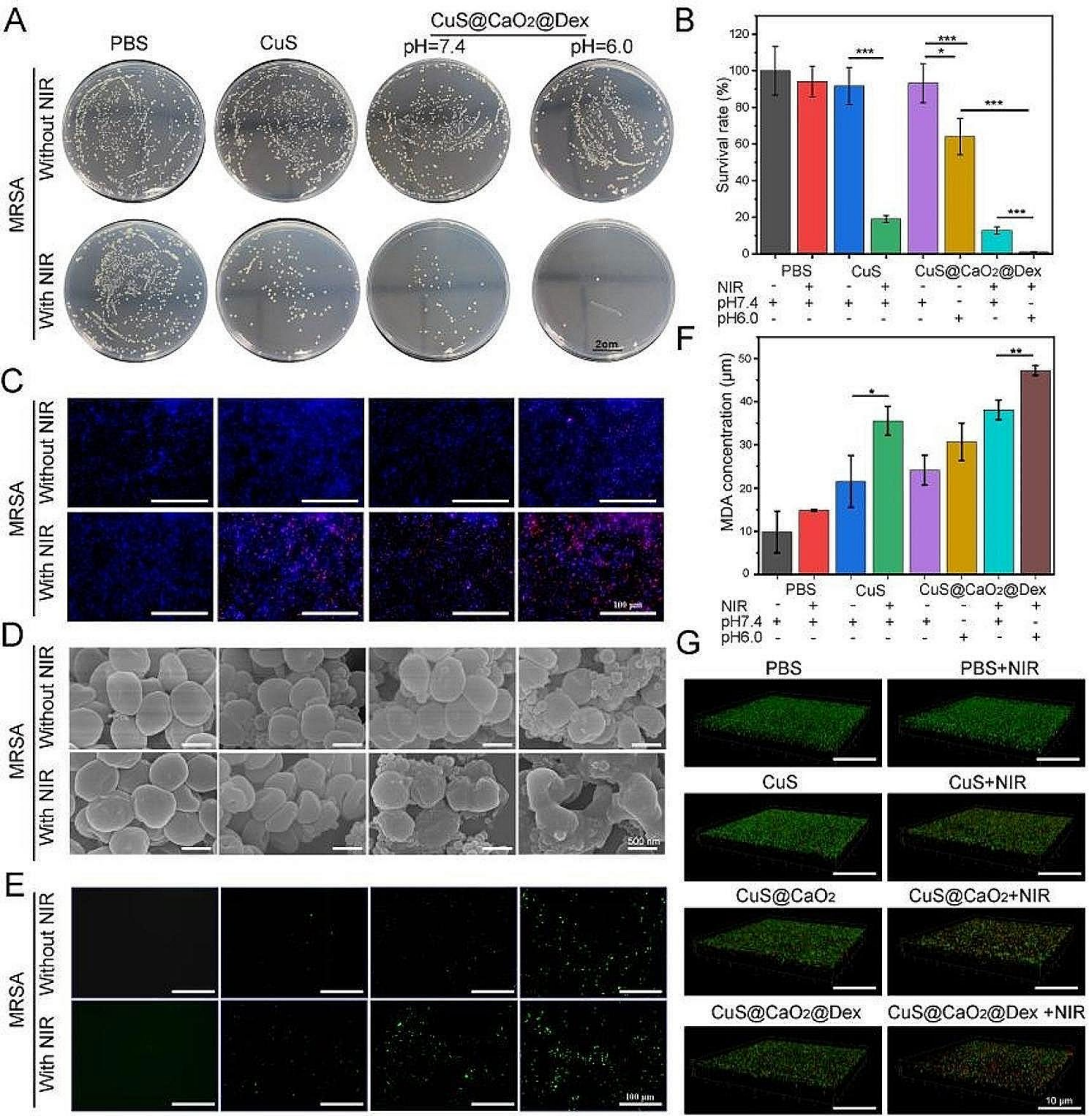
In vitro antibacterial evaluation of CuS@CaO_2_@Dex. (**A**) Pictures of the agar plates of MRSA after different treatments. (**B**) The corresponding survival rate of (**A**). (**C**) Fluorescence staining images of MRSA by DAPI/PI after diverse treatments. (**D**) SEM images of MRSA morphology through different treatments. (**E**) ROS levels of MRSA by DCFH-DA via diverse treatments. (**F**) MDA concentration of MRSA after diverse treatments. (**G**) CLSM images of MRSA biofilms after various treatments

To investigate the synergistic antibacterial effect of CuS@CaO_2_@Dex, MRSA was treated with various nanomaterials. The images of the agar plate and the survival rate were demonstrated in Fig. 4A and B. Similar to the PBS control group, the PBS + NIR group did not cause significant damages to MRSA. The pure CuS group exhibited no significant inhibitory effect on bacterial growth, while the CuS + NIR group demonstrated substantial antibacterial activity through PTT (80.95%). Due to the H_2_O_2_ self-supplying •OH generation, CuS@CaO_2_@Dex exhibited antibacterial activity under slightly acidic condition (CuS@CaO_2_@Dex + pH 6.0, 35.95%), but had no antibacterial activity under neutral conditions (CuS@CaO_2_@Dex + pH 7.4, 6.74%). Even so, the CDT of CuS@CaO_2_@Dex alone was not sufficient to completely eliminate bacteria. When CuS@CaO_2_@Dex was exposed to NIR (CuS@CaO_2_@Dex + pH 7.4 + NIR), its antibacterial efficiency reached 87.24%. With the aid of CDT (CuS@CaO_2_@Dex + pH 6.0 + NIR), the synergistic antibacterial rate further rose to 98.92%, illustrating that the combined CDT/PTT antibacterial effect was significant.

Subsequently, we further investigated the antibacterial property of CuS@CaO_2_@Dex via live/dead staining assays. MRSA was treated with different nanocomposite materials and then stained with both DAPI and PI before fluorescence imaging. As shown in Fig. 4C, the PBS, PBS + NIR, CuS and CuS@CaO_2_@Dex + pH 7.4 groups displayed little red fluorescence (indicating dead bacteria), indicating negligible antibacterial activity. CuS + NIR, CuS@CaO_2_@Dex + pH 6.0, and CuS@CaO_2_@Dex + pH 7.4 + NIR showed the coexistence of red and blue fluorescence in different ratios for MRSA, illustrating varying degrees of bacterial damages produced due to the CDT or NIR effect. More significantly, only CuS@CaO_2_@Dex + pH 6.0 + NIR group presented the most abundant red fluorescence, implying that most of bacteria were eradicated through the synergistic performance of CDT/PTT. These results confirmed the outstanding CDT/PTT synergistic antibacterial activity of CuS@CaO_2_@Dex.

To better identify the antibacterial mechanism of CuS@CaO_2_@Dex, we further explored the morphology and integrity of MRSA. As shown in Fig. 4D, compared with the negligible damages on the bacterial surface of the PBS, PBS + NIR and CuS groups, the morphology of MRSA in CuS + NIR and CuS@CaO_2_@Dex groups exhibited different degrees of damage mediated by CDT or NIR effect. Notably, due to the synergistic treatment of CDT/PTT, MRSA in CuS@CaO_2_@Dex + pH 6.0 + NIR group showed the collapse of bacterial skeleton structure and the lysis of bacterial membranes, illustrating severe structural damage and destruction of the bacteria. TEM (Fig. S8) also showed the same experimental phenomenon. Furthermore, we examined ROS levels in bacterial cells after various treatments. As shown in Fig. 4E, PBS, PBS + NIR and CuS groups had little or no green fluorescence signals, indicating minimal ROS levels in the cells. Simultaneously, various levels of green fluorescence in bacteria were found in CuS + NIR, CuS@CaO_2_@Dex + pH 7.4, CuS@CaO_2_@Dex + pH 6.0 and CuS@CaO_2_@Dex + pH 7.4 + NIR groups, illustrating that NIR or CDT induced nanomaterials to generate varying amounts of ROS. Notably, the fluorescence intensity of ROS in the CuS @CaO_2_@Dex + pH 6.0 + NIR group was the strongest, illustrating that the combined CDT/PTT treatment of CuS@CaO_2_@Dex could produce a significant amount of ROS in bacteria, leading to bacterial lysis or death. We further assessed lipid peroxidation by measuring the level of intracellular malondialdehyde (MDA). The concentration of MDA in CuS + NIR was significantly higher than CuS due to NIR irradiation. Besides, CuS@CaO_2_@Dex + pH 6.0 + NIR had higher MDA level than CuS@CaO_2_@Dex + pH 7.4 + NIR, mainly because of the effect of CDT. These results suggested that CuS@CaO_2_@Dex could induce membrane lipid peroxidation (Fig. 4F).

### Antibiofilm activity in vitro

Biofilms contain complex EPS that protect biofilm bacteria from host immune cells and hinder antibiotic penetration. In this study, dextran was coated on the surface of nanocomposites to promote its permeation into biofilm. We investigated the ability of CuS@CaO_2_@Dex to permeate into and eliminate biofilms by crystal violet staining. PBS, PBS + NIR and CuS groups all showed little influence on biofilm removal (Fig. S9A). In contrast, 150 µg mL^− 1^ CuS@CaO_2_@Dex exhibited more efficient killing activities against MRSA biofilm than CuS@CaO_2_. Moreover, under 1064 nm NIR laser (0.75 W cm^− 2^), CuS@CaO_2_@Dex + NIR had the better ability of biofilms elimination than CuS@CaO_2_ + NIR. These results illustrated that dextran coatings evidently enhanced penetration into biofilm and the effect of biofilm removal. Furthermore, CuS@CaO_2_@Dex + NIR treatment had the greatest degree of damage to the biofilm, attributed to the synergic sterilization activity of CDT/PTT. Subsequently, the biofilms with different treatments were quantified by determining the absorbances at 590 nm. As seen in Fig. S9B, CuS@CaO_2_@Dex + NIR group, owing to the synergistic activity of CDT/PTT, showed lower remaining biofilm (41.8%) than CuS@CaO_2_@Dex (66.0%, CDT). Interestingly, CuS@CaO_2_@Dex (66.0%) had lower remaining biofilm than CuS@CaO_2_ alone (90.5%). Similar experimental results also appeared in CuS@CaO_2_@Dex + NIR (41.8%), with significantly lower biofilm residues than in CuS@CaO_2_ + NIR (63.7%), which was possibly attributed to the great penetration of CuS@CaO_2_@Dex. These results confirmed the effective biofilm eradication of CuS@CaO_2_@Dex due to its excellent penetration and CDT/PTT combined treatment.

To further investigate the capacity of CuS@CaO_2_@Dex to permeate and eliminate biofilms, MRSA biofilms after different treatments were stained with SYTO9, and PI, then visualized by CLSM. As shown in Fig. 4G, PBS, PBS + NIR and CuS groups showed integrated and dense the membranes with almost all green fluorescence (indicating live bacteria) and negligible red fluorescence (indicating dead bacteria). This illustrated that the biofilm could not be disrupted under these conditions. The presence of dextran (CuS@CaO_2_@Dex) significantly enhanced the dispersal of the biofilm with laser irradiation or not, mainly correlated with the great dispersibility of CuS@CaO_2_@Dex. Additionally, the presence of dextran, acting as bacterial polysaccharide, could enhance the biocompatibility of CuS@CaO_2_@Dex and further increase the affinity between CuS@CaO_2_@Dex and biofilms, providing another explanation for promoting the penetrability of CuS@CaO_2_@Dex. The CuS@CaO_2_@Dex + NIR group exhibited the strongest red fluorescence, and the density and thickness of the MRSA biofilm sharply decreased, which indicated that most bacteria were killed, and the biofilm structure was significantly disrupted and eliminated. These results indicated that the synergistic action of CuS@CaO_2_@Dex by CDT/PTT treatment and the enhanced penetration by dextran could significantly eliminate MRSA biofilm. Thus, CuS@CaO_2_@Dex could potentially eradicate bacteria and clear biofilms in infected wounds, providing a promising avenue for the development of antibiofilm agents.

### In vivo antibacterial performance

**Fig. 5:**
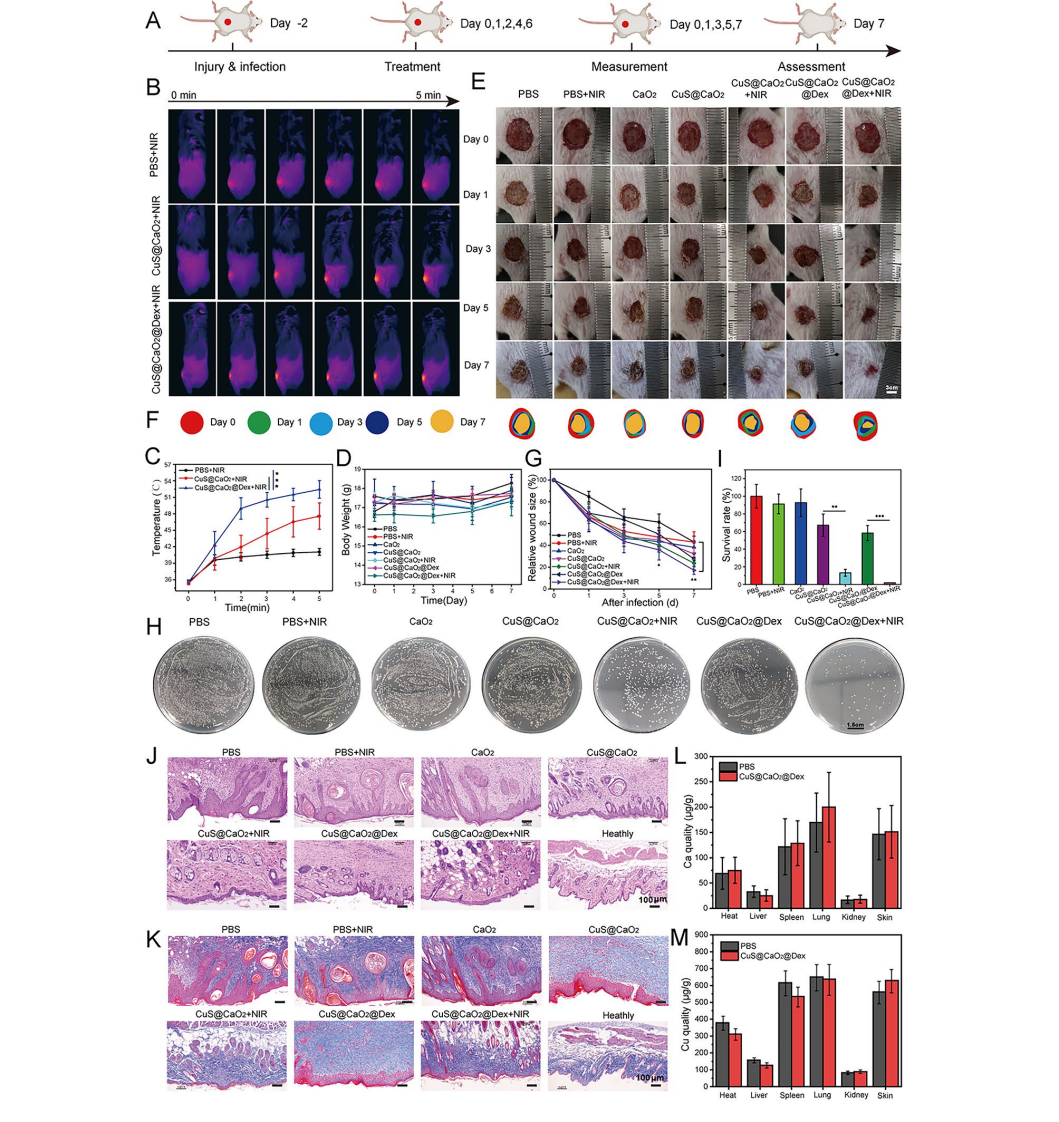
Antibacterial evaluation of CuS@CaO_2_@Dex in vivo. (**A**) Schematic diagram for the construction of the mice wound infection model and the subsequent treatment strategies. (**B**) Evaluation of photothermal performance of CuS@CaO_2_@Dex in the wound. (**C**) Photothermal curves of CuS@CaO_2_@Dex. (**D**) The mice weight during the treatment period. (**E**) Pictures of infected wound sites after varying treatments. (**F**) Traces of wound closure. (**G**)The relative area of the wound after various treatments and different time. (**H**) Pictures of the agar plates of MRSA with various treatments. (**I**) The corresponding survival rate after diverse treatments. (**J**) and (**K**) H&E and Masson staining of wound sites after various treatments. (**L**) and (**M**) The content of calcium and copper in the major organs and skin of mice

Considering the excellent in vitro antibacterial and antibiofilm ability, we established a mouse wound infection model to investigate the in vivo antibacterial properties of CuS@CaO_2_@Dex. Freshly prepared skin wounds were inoculated with MRSA suspensions (100 µL, 1 × 10^8^ CFU mL^− 1^) and cultured for 48 h to build the biofilm infection model (Fig. 5A). The temperature variation in infected wound treated with various nanomaterials were visualized with a thermal image under the 1064 nm NIR laser (0.75 W cm^− 2^). Interestingly, the temperature of CuS@CaO_2_@Dex group in the infected wounds rose from 35.6 to 52.4 °C under the existence of NIR laser irradiation, significantly higher than increase observed in the CuS@CaO_2_ group (increased to 47.6 °C) (Fig. 5B and C). In contrast, the PBS control group showed only a slight temperature increase (from 35.6 to 41.1 °C). We hypothesized that dextran-coated nanomaterials effectively penetrated and bound to the biofilm in the infected wounds, anchoring CuS@CaO_2_@Dex and significantly increasing the temperature in the wounds. However, CuS@CaO_2_ + NIR and PBS + NIR lacking dextran, could diffuse to other parts of the wound, resulting in a weaker photothermal effect within the wound. To assess the potential of NIR laser irradiation causing skin burns, CuS@CaO_2_@Dex was administered via subcutaneous injection, followed by an exposure for 5 min to 1064 NIR laser (0.75 W cm^− 2^). Normal mice and normal mice + NIR were employed as control groups. Subsequent to NIR laser exposure, blood samples were taken out for blood biochemical and blood biochemical testing. Meanwhile, mouse skin and internal organs (heart, liver, spleen, lung, and kidney) were collected at the end of treatment, and H&E and TdT-mediated dUTP nick end labeling (TUNEL) assays were further performed to further study skin burns. As depicted in Fig. S10A, after injection of CuS@CaO_2_@Dex, the biochemical parameters of mice were not significantly different from those of healthy mice, such as aspartate aminotransferase (AST), alanine aminotransferase (ALT), blood urea nitrogen (BUN) and creatinine (CREA), indicating no side effects on liver and kidney function. Routine blood indicators showed that injection of nanocomplexes did not cause inflammatory reactions in mice (Fig. S10B). The major organs showed negligible histological damages (Fig. S10C). TUNEL staining showed no large apoptosis in skin cells (Fig. S10D). These results clearly indicated that CuS@CaO_2_@Dex exhibited desirable photothermal properties in vivo.

During the treatment, the wounds of each group were photographed at daily intervals. To facilitate an accurate comparison of wound healing among the different treatments, the wound size was systematically recorded. Notably, in comparison to the other groups, the relative wound area of CuS@CaO_2_@Dex + NIR group decreased to 17.2% at the conclusion of the treatment period, illustrating an optimal healing process attributable to the synergistic antibacterial effects of CDT/PTT (Fig. 5E and G). Simultaneously, the other groups exhibited varying degrees of reduction in the rate of wound healing, with the PBS group demonstrating the slowest rate (43.1%). The antibacterial activity of various treatments at the wound sites was investigated by the plate counting method. In the later stage of treatment, the bacterial burden of CuS@CaO_2_@Dex + NIR group was the lowest than other groups, achieving an antibacterial efficiency of 98.2% (Fig. 5H and I). In contrast, other treatment groups displayed varying levels of bacterial residues during the same period, with the PBS group showing the highest number of bacterial residues. These results underscore the effectiveness of combined CDT/PTT treatment in addressing MRSA infection at the wound sites and achieving desirable wound healing. Importantly, the weight of the mice did not exhibit significant variations throughout the treatment duration, indicating that the aforementioned treatments did not adversely impact the normal growth of the mice (Fig. 5D).

Furthermore, after excising the wound skins at the conclusion of the treatment, we conducted H&E assays to gain further insights into wound healing. As observed in Fig. 5J, a higher number of inflammatory cells were evident in PBS, PBS + NIR and CaO_2_ groups, revealing the presence of serious infection and inflammation at the wound sites. Both CuS@CaO_2_ and CuS@CaO_2_@Dex groups exhibited varying degrees of reduction in inflammatory cell infiltration. Notably, the CuS@CaO_2_ + NIR and CuS@CaO_2_@Dex + NIR groups displayed minimal inflammatory cells and demonstrated the formation of a complete epidermal layer, indicative of effective wound healing. Additionally, Masson staining assays were performed to further assess the recovery of skin tissues. As illustrated in Fig. 5K, various treatment groups exhibited substantial collagen deposition (blue) in the wound tissues, except for the PBS, PBS + NIR and CaO_2_ groups. In summary, the constructed CuS@CaO_2_@Dex group had good performance for decreasing inflammatory reaction and promoting wound healing. The metal (calcium and copper) content in the mouse viscera and skin was measured by the flame atomic absorption method on day 7 to explore the degradation of the CuS@CaO_2_@Dex. As shown in Fig. 5L, the calcium content in the mice almost returned to normal levels on day 7, and a similar pattern was observed in the copper content in mice. The experimental results suggest that CuS@CaO_2_@Dex was metabolized and did not accumulate in the major organs.

#### Cytotoxicity assay

Biocompatibility is a crucial consideration for the medical applications of antibacterial agents. Therefore, the biocompatibility of CuS@CaO_2_@Dex, including the cytocompatibility and hemocompatibility, was thoroughly investigated. The results showed that 150 µg mL^− 1^ CuS@CaO_2_@Dex exhibited more than 80% cell viability, indicating no significant cytotoxicity associated with CuS@CaO_2_@Dex (Fig. S11A). Subsequently, a hemolysis assay was conducted to evaluate the hemocompatibility of CuS@CaO_2_@Dex. As presented in Fig. S11B, the supernatant from the CuS@CaO_2_@Dex group appeared colorless and clear, comparable to the results from the PBS group (negative control), and distinct from the water group (positive control), signifying excellent blood compatibility.

Simultaneously, we investigated the safety and the migration ability of fibroblasts for CuS@CaO_2_@Dex using the transwell cell migration experiment. Fibroblasts migration had a significant role in enhancing wound healing by secreting collagen and elastin to form collagen fibers and elastic fibers. As presented in Fig. S12A, the comparison between damaged cells and those treated with CuS@CaO_2_@Dex and CuS@CaO_2_@Dex + NIR revealed a notable increase in fibroblast migration. Additionally, the number of migrating fibroblasts after various treatments was quantified. As shown in Fig. S12B, CuS@CaO_2_@Dex and CuS@CaO_2_@Dex + NIR exhibited a significant enhancement in fibroblast cell migration, which attributed to the presence of copper ions promoting wound healing. Simultaneously, the migratory ability of CuS@CaO_2_@Dex + NIR showed no significantly difference compared to CuS@CaO_2_@Dex, suggesting that NIR irradiation did not affect fibroblast growth and migration. Furthermore, the migration ability of damaged fibroblasts after treatments with CuS@CaO_2_@Dex and CuS@CaO_2_@Dex + NIR did not significantly decline when compared to normal fibroblasts. These findings indicated that CuS@CaO_2_@Dex effectively promoted fibroblast migration, facilitating wound healing without inducing toxicity.

**Fig. 6:**
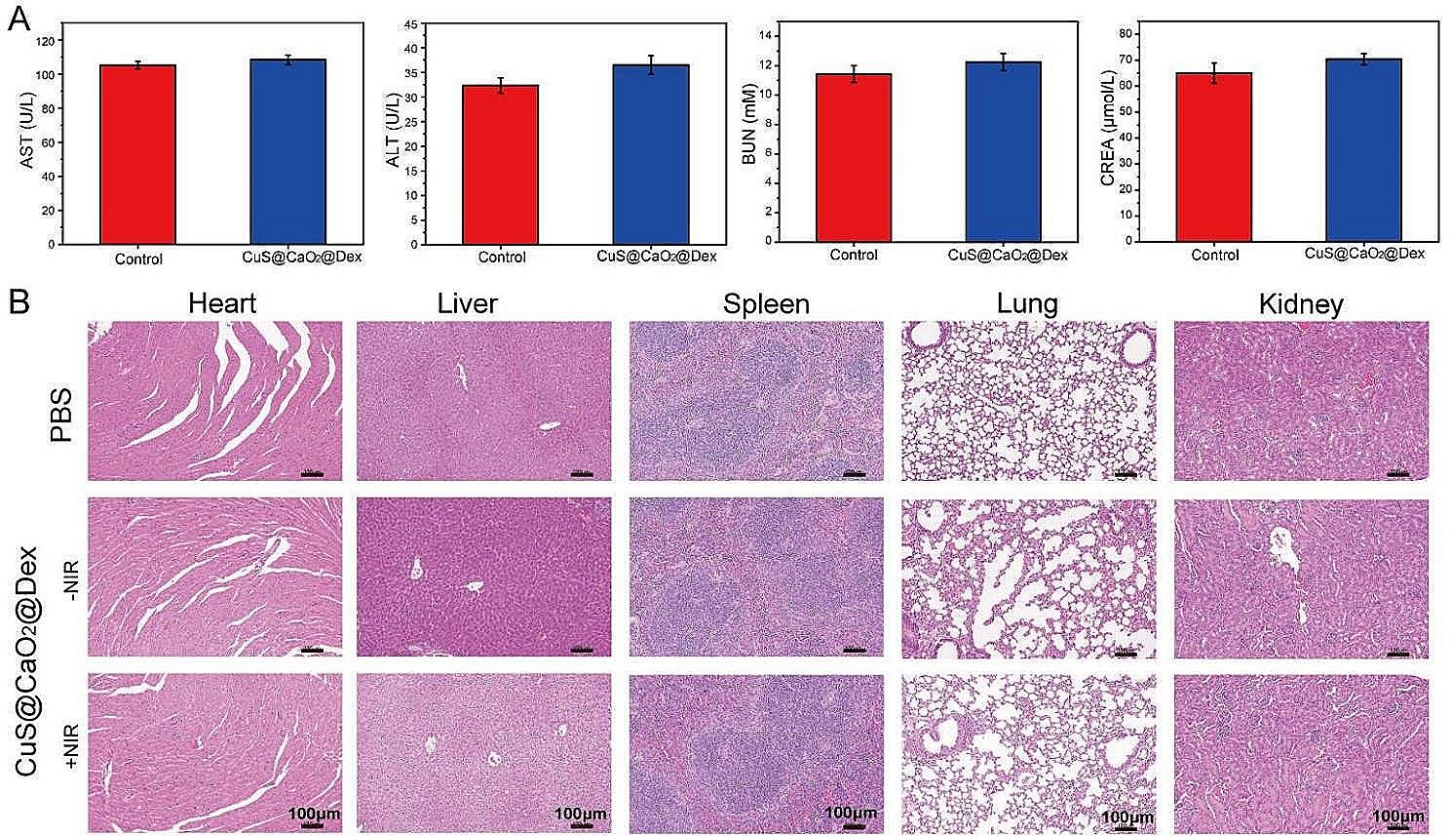
Biosafety assessment of CuS@CaO_2_@Dex. (**A**) The biochemical indicators of mouse blood after different treatments. (**B**) H&E staining of the heart, liver, spleen, lung and kidney tissues

The in vivo biosafety of CuS@CaO_2_@Dex was investigated. A solution of 150 µg mL^− 1^ CuS@CaO_2_@Dex (50 µL) was injected into the mice, which were euthanized after 24 h, and blood was collected for biochemical testing. After 7 days of treatment, the visceral organs (heart, liver, spleen, lung, and kidney) of the mice were harvested and subjected to H&E staining. As depicted in Fig. 6A, after injection of CuS@CaO_2_@Dex, the biochemical parameters of mice were not significantly different from those of healthy mice, such as aspartate aminotransferase (AST), alanine aminotransferase (ALT), blood urea nitrogen (BUN) and creatinine (CREA), indicating no side effects on liver and kidney function. In addition, the major organs exhibited negligible histological damage between the CuS@CaO_2_@Dex and healthy groups (Fig. 6B).

## Conclusion

In summary, we successfully developed a dextran-coated nanoplatform, denoted as CuS@CaO_2_@Dex, with H_2_O_2_ self-supply and GSH depletion for synergistic CDT/PTT therapy aimed at biofilms elimination. Operative under the acidic condition, CuS@CaO_2_@Dex underwent rapid degradation, releasing H_2_O_2_ and generating highly toxic •OH through a Fenton-like reaction. Additionally, the nanoplatform exhibited excellent photothermal performance, accelerating the efficacy of CDT. Furthermore, CuS@CaO_2_@Dex demonstrated efficient GSH depletion by converting Cu^2+^ to Cu^+^ for enhancing CDT. In vitro experiments demonstrated CuS@CaO_2_@Dex had good antibacterial property against MRSA and effective elimination of mature MRSA biofilms through synergistic CDT/PTT. The introduction of dextran coating exhibited evidently enhanced elimination ability of CuS@CaO_2_@Dex due to significantly promoted the penetrability and biocompatibility. In vivo animal experiments showed that CuS@CaO_2_@Dex had remarkable antibacterial membrane properties and optimal wound healing process. In conclusion, CuS@CaO_2_@Dex represents a promising bactericidal nanoplatform, combining CDT and PTT activities, with potential applications in the clinical treatment of biofilm-associated infectious diseases.

### Electronic supplementary material

Below is the link to the electronic supplementary material.

Supplementary Material 1

## Data Availability

No datasets were generated or analysed during the current study.
