# Supplementary material for: H2O2 self-supplying and GSH-depleting nanosystem for amplified NIR mediated-chemodynamic therapy of MRSA biofilm-associated infections
Source: J Nanobiotechnology. 2024 Mar 16;22:117. doi: 10.1186/s12951-024-02350-6 (PMC10943804; doi:10.1186/s12951-024-02350-6)
Supplement: Supplementary file 1 — Supplementary Material 1 [file 12951_2024_2350_MOESM1_ESM.docx]

Supporting Information

**H_2_O_2_ self-supplying and GSH-depleting nanosystem for amplified NIR mediated-chemodynamic** **therapy of** **MRSA biofilm-associated infections**

Yulan Zhao ^1^ ^†^, Yang Wu ^1 †^, Quan Xu ^2^, Yi Liu ^2^, Zhiyong Song ^2^, and Heyou Han ^1, 2^*

† These authors contributed equally to this work

^1^ National Key Laboratory of Agricultural Microbiology, College of Life Science and Technology, Huazhong Agricultural University, Wuhan 430070, China

^2^ National Key Laboratory of Agricultural Microbiology, College of Chemistry, Huazhong Agricultural University, Wuhan 430070, China

^*^**Correspondence:** Heyou Han

**E-mail:** [hyhan@mail.hzau.edu.cn](mailto:hyhan@mail.hzau.edu.cn)


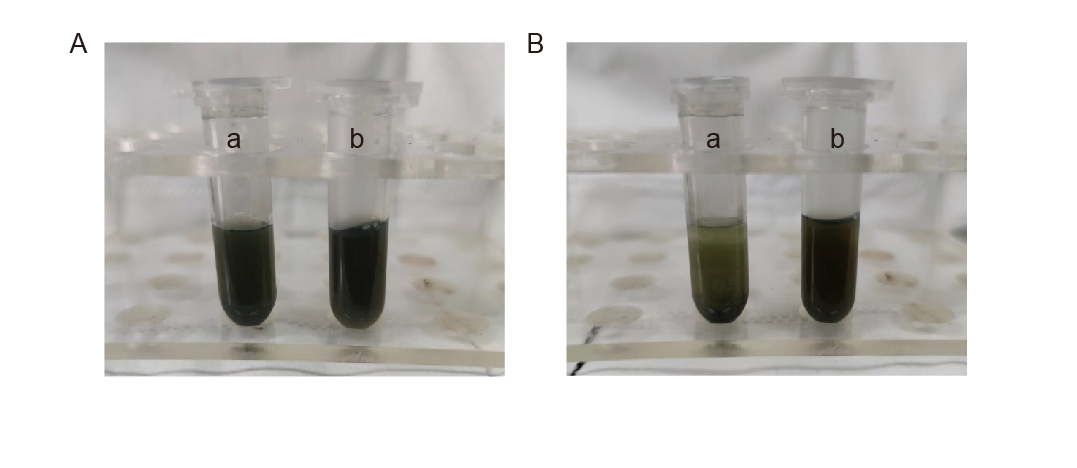


**Fig. S1** Photographs of CuS@CaO_2_ (a) and CuS@CaO_2_@Dex (b) in PBS before (A) and after 2 h (B).


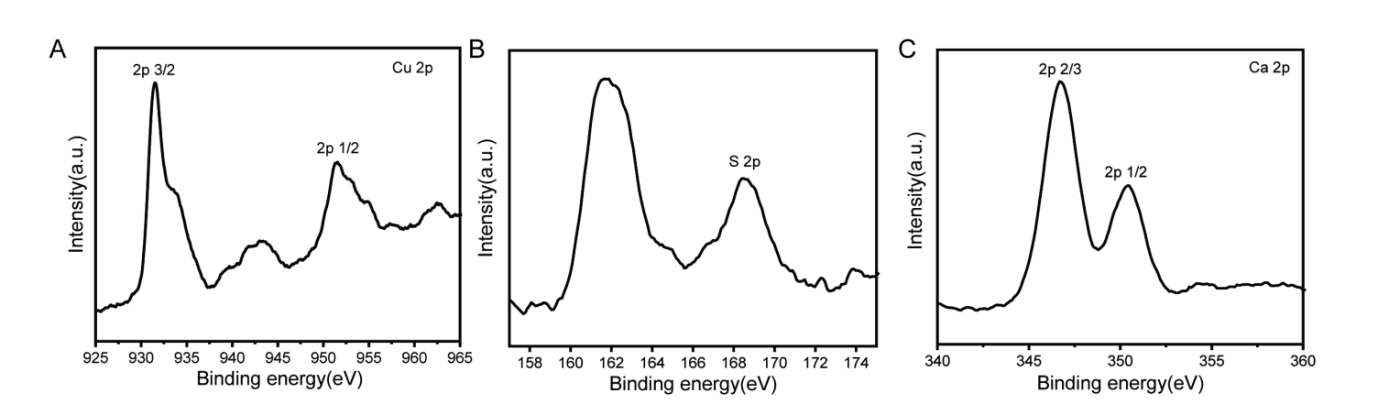


**Fig. S2** High-resolution XPS scan of Cu 2p, S 2p and Ca 2p obtained from CuS@CaO_2_@Dex.


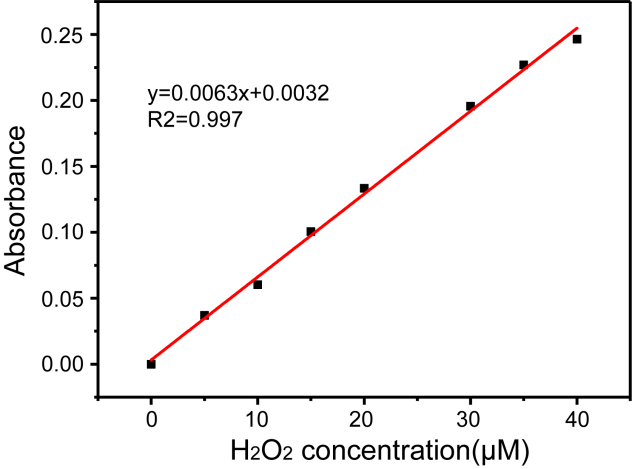


**Fig. S3** Standard curve for the detection of H_2_O_2_ (R^2^=0.997).


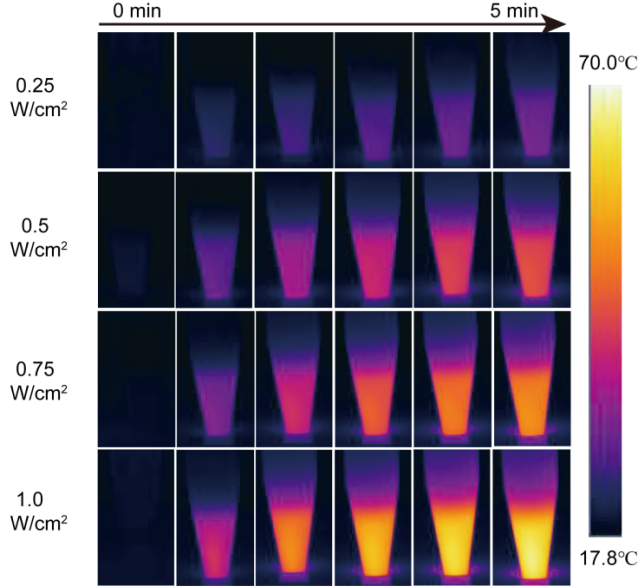


**Fig. S4** Thermal images of CuS@CaO_2_@Dex under different laser power densities (200 μg/mL).


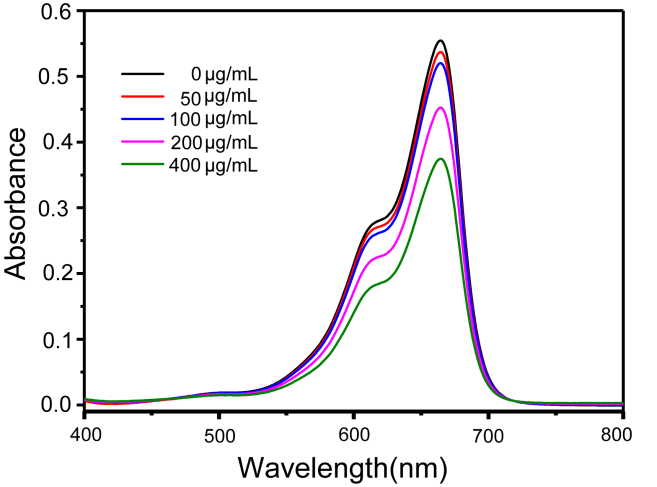


**Fig. S5** UV-vis absorbance spectra of MB degradation in the presence of various concentrations of CuS@CaO_2_@Dex at pH 6.0 for 30 min.


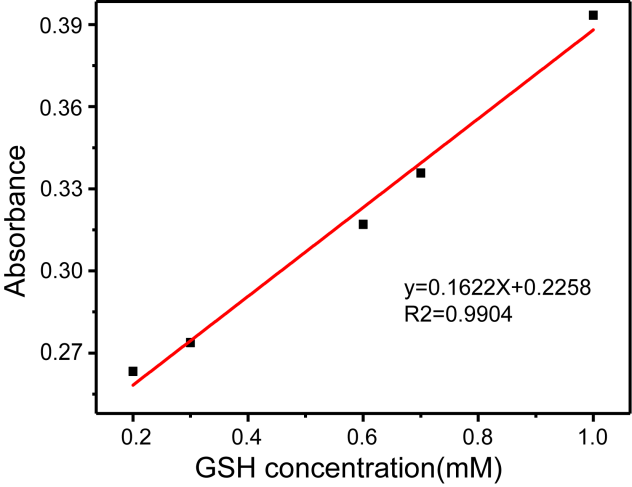


**Fig. S6** Standard curves of GSH at the peak of 407 nm using DTNB.


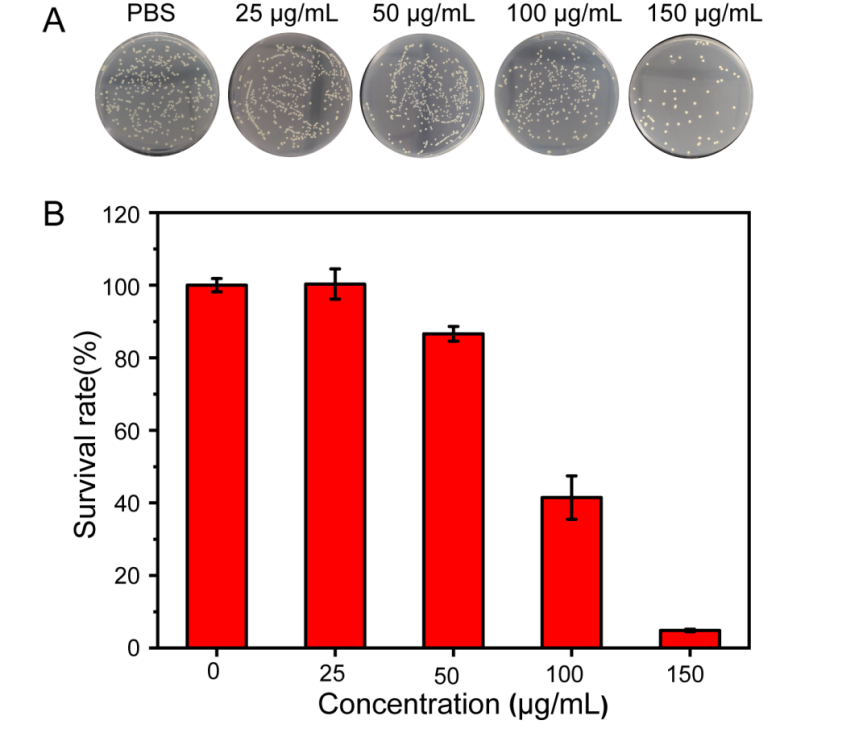


**Fig. S7** Photographs of the agar plates of MRSA treated with CuS@CaO_2_@Dex with different concentrations under 1064 nm laser irradiation and the corresponding survival rate.


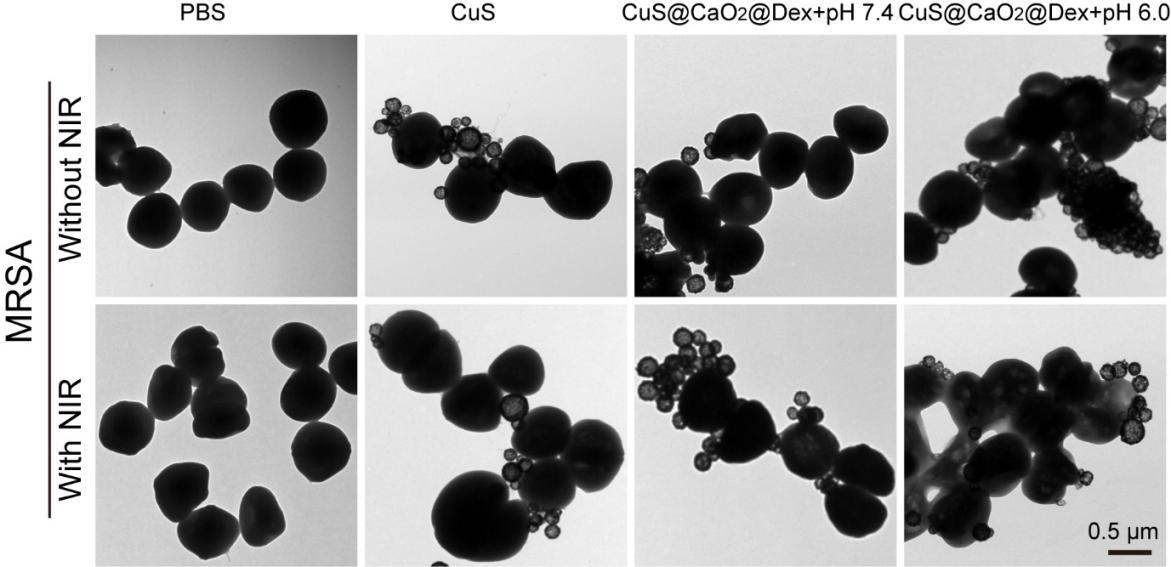


**Fig. S8** TEM images of MRSA morphology after various treatments.


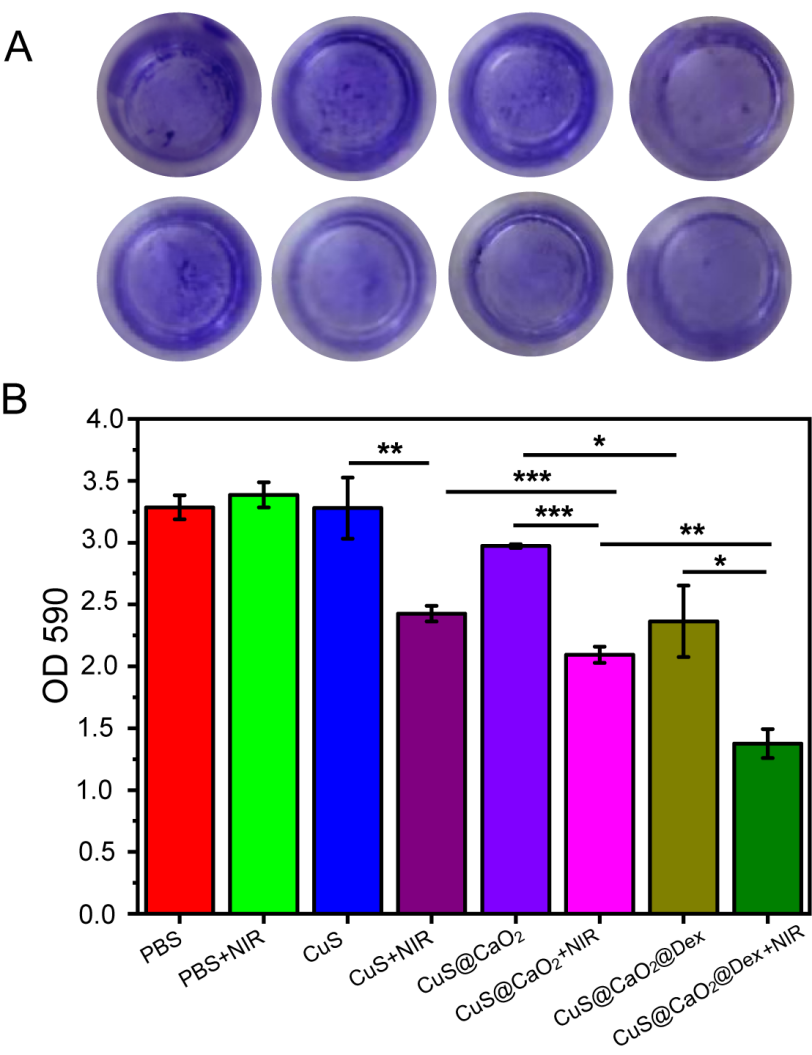


**Fig. S9** The effect of CuS@CaO_2_@Dex on the biofilm. (A) The staining picture of crystal violet on the biofilms after different treatments. (B) The quantitative picture of biofilms after different treatments.


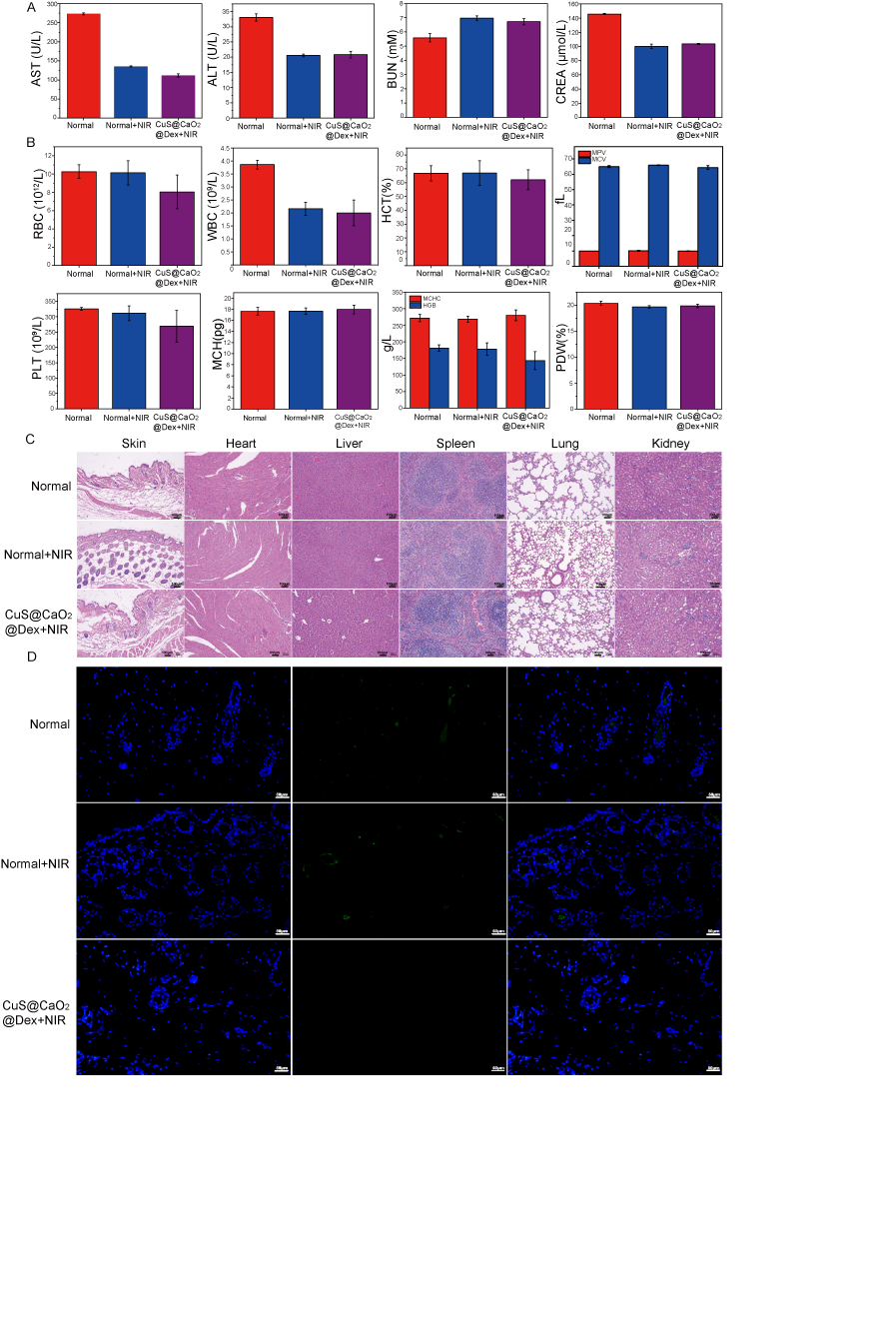


**Fig.** **S10** Biosafety assessment of NIR laser irradiation causing skin burns. (A) The biochemical indicators of mouse blood after different treatments. (B) blood panel analysis test of mice after different treatments. (C) H&E staining of the heart, liver, spleen, lung and kidney tissues. (D) TUNEL analysis after different treatments.


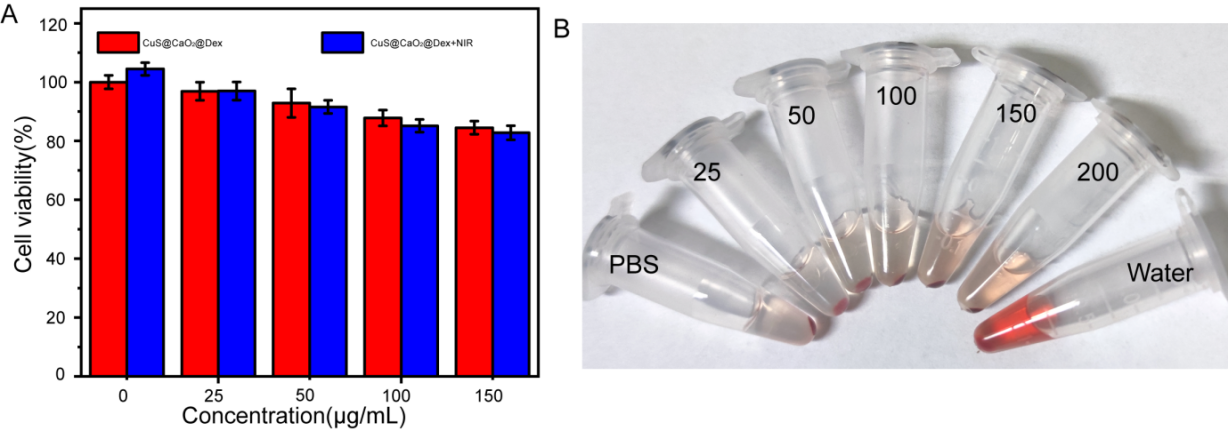


**Fig.** **S11** Biocompatibility. (A) Relative viability of LO_2_ with different concentration of CuS@CaO_2_@Dex. (B) Photographic images of mice blood incubated with a series of concentrations of CuS@CaO_2_@Dex.


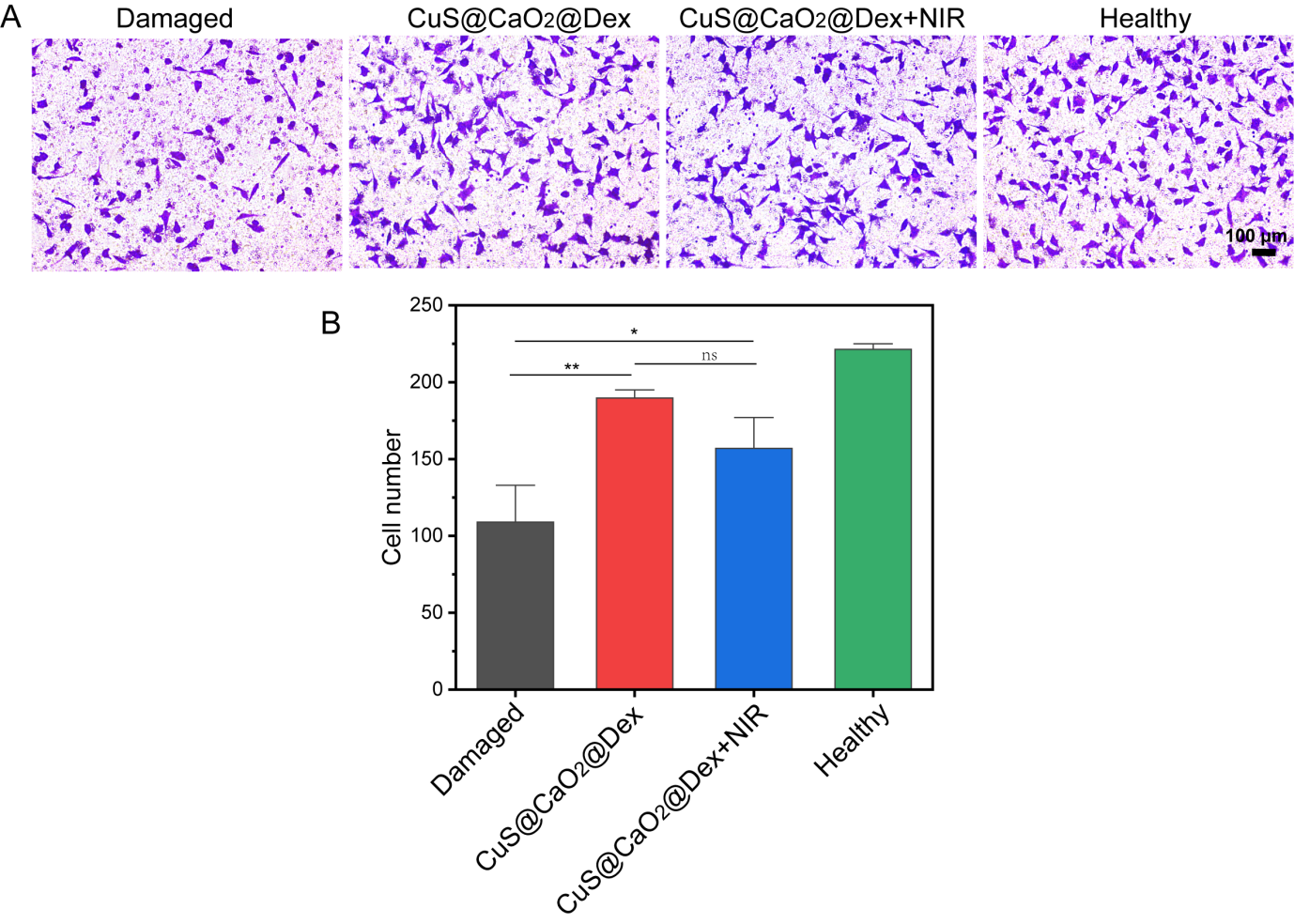


**Fig. S12** Transwell cell migration experiment of fibroblasts after various treatments.
